# DNA methylation regulator-mediated modification patterns and tumor microenvironment characterization in glioma

**DOI:** 10.18632/aging.204291

**Published:** 2022-09-21

**Authors:** Haitao Luo, Minhua Ye, Yan Hu, Miaojing Wu, Mengqi Cheng, Xingen Zhu, Kai Huang

**Affiliations:** 1 Department of Neurosurgery, The Second Affiliated Hospital of Nanchang University, Nanchang, Jiangxi Province, China; 2 Jiangxi Key Laboratory of Neurological Tumors and Cerebrovascular Diseases, Nanchang, Jiangxi Province, China; 3 East China Institute of Digital Medical Engineering, Shangrao, Jiangxi Province, China; 4 Institute of Neuroscience, Nanchang University, Nanchang, Jiangxi Province, China; 5 Department of Obstetrics and Gynecology, Suizhou Central Hospital, Hubei University of Medicine, Suizhou, Hubei Province, China

**Keywords:** DNA methylation, immune phenotypes, tumor mutation burden, immunotherapy, glioma

## Abstract

Growing evidences indicate DNA methylation plays a crucial regulatory role in inflammation, innate immunity, and immunotherapy. However, the overall landscape of various DNA methylation regulatory genes and their relationship with the infiltration of immune cells into the tumor microenvironment (TME) as well as the response to immunotherapy in gliomas is still not clear. Therefore, we comprehensively analyzed the correlation between DNA methylation regulator patterns, infiltration of immune cell-types, and tumor immune response status in gather glioma cohorts. Furthermore, we calculated the DNA methylation score (DMS) for individual glioma samples, then evaluated the relationship between DMS, clinicopathological characteristics, and overall survival (OS) in patients with gliomas. Our results showed three distinct DNA methylation regulator patterns among the glioma patients which correlated with three distinct tumor immune response phenotypes, namely, immune-inflamed, immune-excluded, and immune desert. We then calculated DMS for individual glioma samples based on the expression of DNA methylation-related gene clusters. Furthermore, DMS, tumor mutation burden (TMB), programmed death 1 (PD-1) expression, immune cell infiltration status in the TME, and Tumor Immune Dysfunction and Exclusion (TIDE) scores were associated with survival outcomes and clinical responses to immune checkpoint blockade therapy. We also validated the predictive value of DMS in two independent immunotherapy cohorts. In conclusion, our results demonstrated that three DNA methylation regulator patterns that correlated with three tumor immune response phenotypes. Moreover, we demonstrated that DMS was an independent predictive biomarker that correlated with survival outcomes of glioma patients and their responses to immunotherapy therapeutic regimens.

## INTRODUCTION

DNA methylation is of the most extensively studied epigenetic modifications that plays a crucial role in the regulation of several biological processes; abnormal changes in DNA methylation are associated with several human diseases, including cancers [1–3]. DNA methylation profiling is an important analytical tool for classifying patients with brain tumors into various subgroups [4].

Glioblastoma (GBM) is the most common primary brain tumor in adults [5]. Despite availability of standard treatment strategies such as surgery, chemotherapy, and radiotherapy, the overall survival (OS) of GBM patients is less than 14 months [6–9]. Therefore, there is an urgent need to identify and characterize more effective therapeutic strategies for improving the survival outcomes of GBM patients.

Immune checkpoint blockade (ICB) therapies have been approved for many malignant tumors, including GBM, but their efficacy is observed in less than 20% of the patients [10–15]. Tumor mutation burden (TMB) and programmed death 1 (PD-1) are potential biomarkers for identifying patients that could benefit from PD-L1 blockade-based immunotherapy [16]. Several studies have shown that DNA methylation regulatory proteins influence the effectiveness of immunotherapy in patients with malignant cancers [17–19]. Wu et al. reported that TET1 expression positively correlated with the infiltration of immune cells in breast cancer [20]. Xu et al. reported that TET2 activity influenced the effectiveness of anti-PD-L1 therapy in solid cancers through the IFN-γ-JAK/STAT signaling pathway [21].

In this study, we comprehensively analyzed the correlations between DNA methylation regulator patterns, characteristics of infiltration of the immune cell types into the tumor microenvironment (TME), and response to ICB therapies using clinicopathological and transcriptome information from five independent glioma datasets. We also constructed a risk score system based on the DNA methylation status of the glioma samples and analyzed if the DNA methylation score (DMS) accurately predicted clinical responses to ICB therapy using two immunotherapy datasets.

## RESULTS

### Landscape of genetic variations in the DNA methylation regulatory genes in glioma

**Figure 1:**
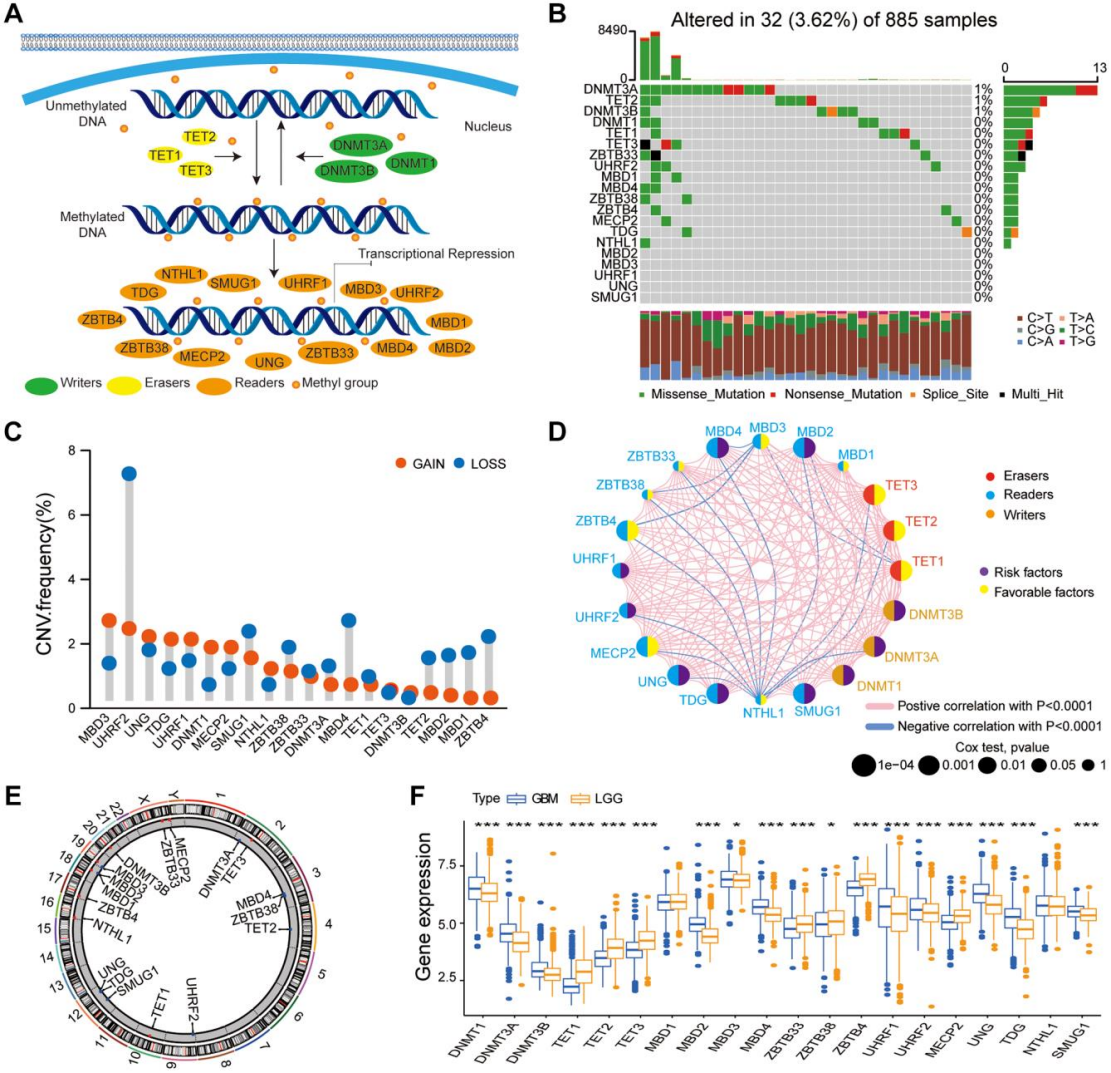
**Multi-omics landscape of the DNA methylation regulators in glioma.** (**A**) The summary of 20 DNA methylation regulators and their molecular functions in mediating the dynamic reversible process of DNA methylation. (**B**) Mutation frequency of the 20 DNA methylation regulators based on TCGA glioma dataset. Each column represents a single glioma samples. (**C**) The CNV frequency of DNA methylation regulators based on the TCGA glioma dataset. Note: gain, red; loss, blue. (**D**) PPI network of the 20 DNA methylation regulators. Size of the node denotes the number of proteins. (**E**) Circos plots illustrating the chromosomal locations of the CNV alternations in 20 DNA methylation regulatory genes. (**F**) Boxplot shows the expression levels of the 20 DNA methylation regulators in patients with LGG and GBM bases on the gather glioma cohort. Note: ns *P* > 0.05; ^*^P < 0.05; ^**^P < 0.01; ^***^P < 0.001.

We selected 20 DNA methylation regulators, containing 3 writers, 3 erasers, and 14 readers after literature survey [22, 23]. The DNA methylation regulatory proteins that regulate the dynamic process of DNA methylation and demethylation and the underlying molecular mechanisms are shown in Figure 1A. The transcriptome and clinicopathological data of the 2228 patients from the TCGA, CGGA1, CGGA2, GSE16011, and GSE108474 glioma datasets, is summarized in Supplementary Table 1.

We then analyzed tumor somatic mutations and copy number variations (CNV) in these DNA methylation regulatory genes in the glioma samples. Somatic mutations in the DNA methylation regulatory genes were observed in only 32 patients; tumor mutation burden (TMB) rate of the 20 regulators was < 1% in the glioma patients (Figure 1B). We observed co-occurrence of mutations in some DNA methylation regulatory genes (MBD4 and TET2; MBD4 and DNMT1; TET3 and DNMT3A; ZBTB33 and DNMT1), mutation-exclusive phenomenon was not observed in any of the 20 DNA methylation regulators (Supplementary Figure 1B). The somatic mutation frequency of glioma patients among DNA methylation regulators was low (<1%), which revealed the different functions of these regulators were not caused by genetic alterations. CNV analysis in the glioma samples demonstrated high frequency of amplifications in MBD3, TDG, UHRF1, DNMT1, and MECP2, and copy number deletions in UHRF2, SMUG1, ZBTB38, MBD4, MBD2, TET2, MBD1, and ZBTB4 (Figure 1C). Figure 1E shows the chromosomal locations of the CNV alternations in these DNA methylation regulatory genes. PPI network analysis showed co-expression of DNA methylation regulatory genes belonging to the same functional group as well as association between writers, erasers, and readers (Figure 1D, Supplementary Table 2). Univariate Cox regression analysis showed that DNMT1, DNMT3A, DNMT3B, MBD2, MBD4, UHRF2, MECP2, UNG, and SMUG1 were potential risk genes, whereas TET1, TET2, TET3, ZBTB4, and MECP2 were potential protective genes in glioma (Supplementary Figure 1C). The expression of 11 DNA methylation regulators (DNMT1, DNMT3A, DNMT3B, MBD2, MBD3, MBD4, UHRF1, UHRF2, UNG, TDG, and SMUG1) was significantly higher in the glioblastoma (GBM) compared to the low-grade glioma (LGG), whereas the expression of 7 DNA methylation regulators (TET1, TET2, TET3, ZBTB33, ZBTB38, ZBTB4, and MECPE) was significantly lower in the GBM (Figure 1F). We performed the immunohistochemistry assay to validate the bioinformatics results, and the assay revealed that three selected DNA methylation regulators (DNMT3A, TET3, and UNG) were expressed to different extents in normal brain tissue (NBT) and LGG (Supplementary Figure 2A–2C), in accord with the bioinformatics results.

Our results indicated most DNA methylation regulators (DNMT1, MBD3, UHRF1, and TDG) with amplificated CNV demonstrated markedly higher expression in GBM, which revealed that the alterations of CNV could be an important element resulting in perturbations on the DNA methylation regulators expression in glioma. This suggested crucial roles for these 20 DNA methylation regulatory genes in glioma progression.

### The relationship between DNA methylation regulator expression, prognosis, and tumor immune characteristics in gliomas

Next, we investigated the role of these DNA methylation regulators in the TME. Spearman’s correlation analysis showed that the expression of UNG, ZBTB33, MECP2, and DNMT3A genes was positively associated with the proportion of several immune cell types in the glioma (Supplementary Figure 3A). Because UNG expression was positively associated with naïve B cells, we systematically investigated the biological functions of UNG in the TME. Kyoto Encyclopedia of Genes and Genomes (KEGG) analysis showed that the high UNG expression group was enriched in several tumor-related pathways, such as p53 signaling pathway, TNF signaling pathway, DNA replication, focal adhesion, NF-kB signaling pathway, and Human T-cell leukemia virus 1 infection (Supplementary Figure 3B). Moreover, gliomas with high UNG expression showed significantly higher infiltration of immune cell types such as naïve B cells, delta gamma T cells, M0 macrophages, activated DCs, and activated mast cells compared to gliomas with low UNG expression (Supplementary Figure 3C). Furthermore, correlation between expression of UNG and clinical response to immunotherapy was evaluated in two immunotherapy cohorts, IMvigor210 and GSE78220. Glioma patients with high UNG high expression in the two immunotherapy cohorts showed better prognosis and clinical response (Supplementary Figure 3D–3G).

These results demonstrated association between the expression of DNA methylation regulatory genes and the infiltration of immune cell types into the TME. Our analysis also suggested that UNG regulated the tumor immune microenvironment and is a potential marker for evaluating the efficacy of immunotherapy in glioma patients.

### Characterization of three DNA methylation regulator patterns in the gathered glioma datasets

**Figure 2:**
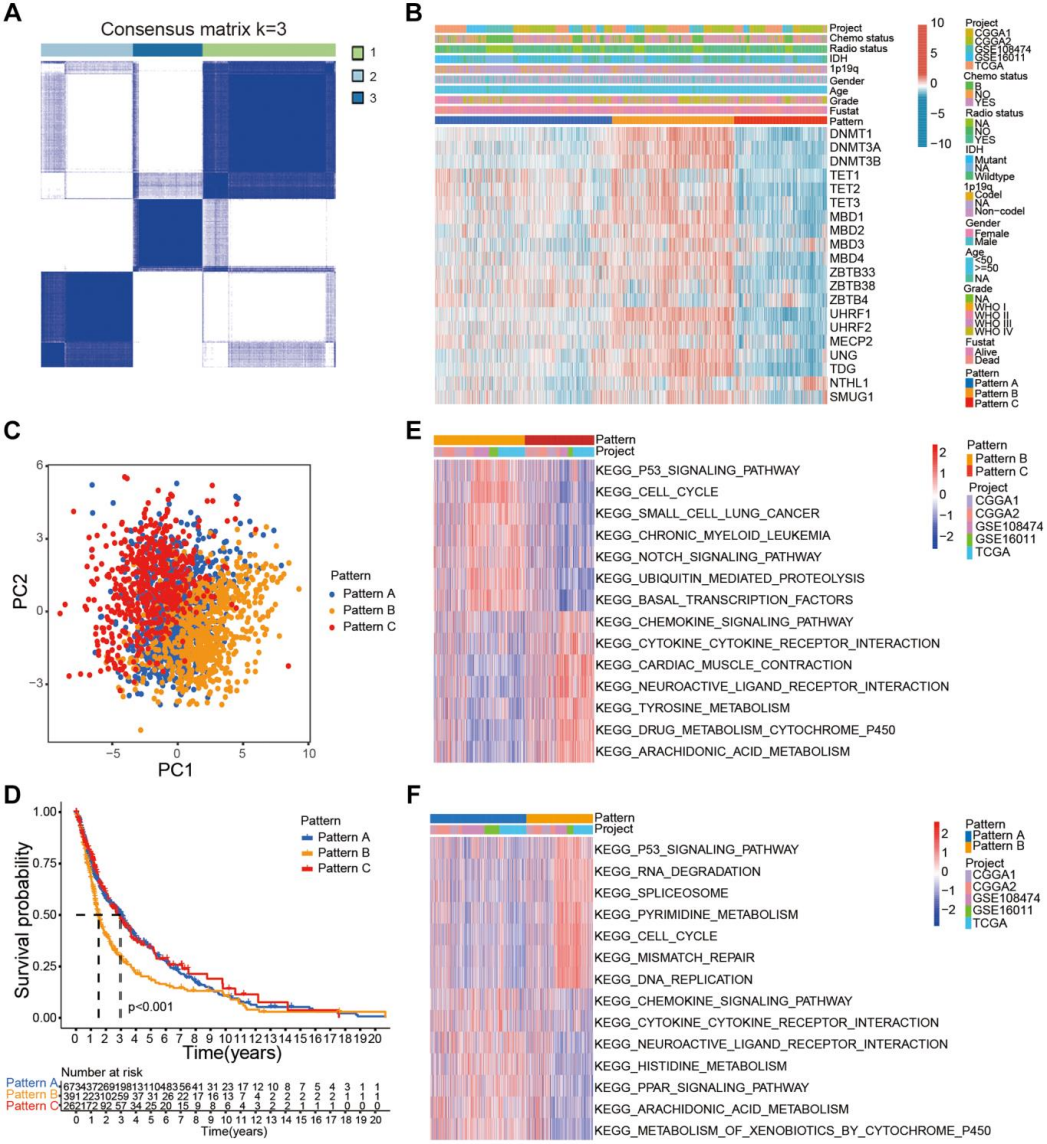
**Characterization of distinct DNA methylation modification patterns in the gathered glioma cohort.** (**A**) Consensus clustering matrix of the gather glioma cohort for k = 3. (**B**) Unsupervised clustering of 20 DNA methylation regulators in the gather glioma cohort. The glioma samples were annotated according to the DNA methylation regulator patterns, glioma grades, 1p19q codeletion status, and IDH status. (**C**) PCA confirmed three distinct patterns based on the expression of the 20 DNA methylation regulator in 2228 glioma samples. (**D**) Kaplan-Meier survival curve analysis showed the OS of glioma samples belonging to the three DNA methylation regulator patterns based on gather glioma cohorts. (**E**, **F**) GSVA analysis shows relatively enriched hallmark gene sets among the three patterns.

DNA methylation regulators might play a crucial role in the heterogeneity of gliomas and the tumor immune microenvironment status [24]. Therefore, to further determine the functions of the DNA methylation regulator, we used the package of ConsensusClusterPlus to identify distinct glioma patient subgroups based on the specific expression patterns of the 20 DNA methylation regulator in the gathered glioma cohort. We performed unsupervised clustering with a K value of 3 and classified glioma samples into three subgroups or modification patterns, namely, pattern A (*n* = 1007), pattern B (*n* = 694), and pattern C (*n* = 527, Figure 2A, Supplementary Figure 4A–4E). The glioma samples with patterns A and C showed high expression of ZBTB4 and NTHL, respectively, whereas, glioma samples with pattern B showed high expression of most DNA methylation regulators (Figure 2B, Supplementary Figure 4F). PCA showed three distinct expression patterns of all 20 DNA methylation regulators, thereby confirming the unsupervised clustering results (Figure 2C). Kaplan-Meier survival curve analysis showed that OS of glioma patients with pattern B were significantly lower compared to those with patterns A and C, probably because majority of the DNA methylation regulators were highly expressed in the pattern B (*P* < 0.001; Figure 2D). Then, we investigated the biological behaviors of the gliomas with these three distinct patterns using GSVA. Patterns A and C demonstrated an immune activation phenotype with enrichment of immune pathways such as the chemokine signaling pathway, cytokine-cytokine receptor interaction, and arachidonic acid metabolism; glioma in pattern B were enriched in stromal activation-related pathways, such as p53 signaling pathway, RNA degradation, cell cycle, and DNA replication (Figure 2E, 2F; Supplementary Table 3).

**Figure 3:**
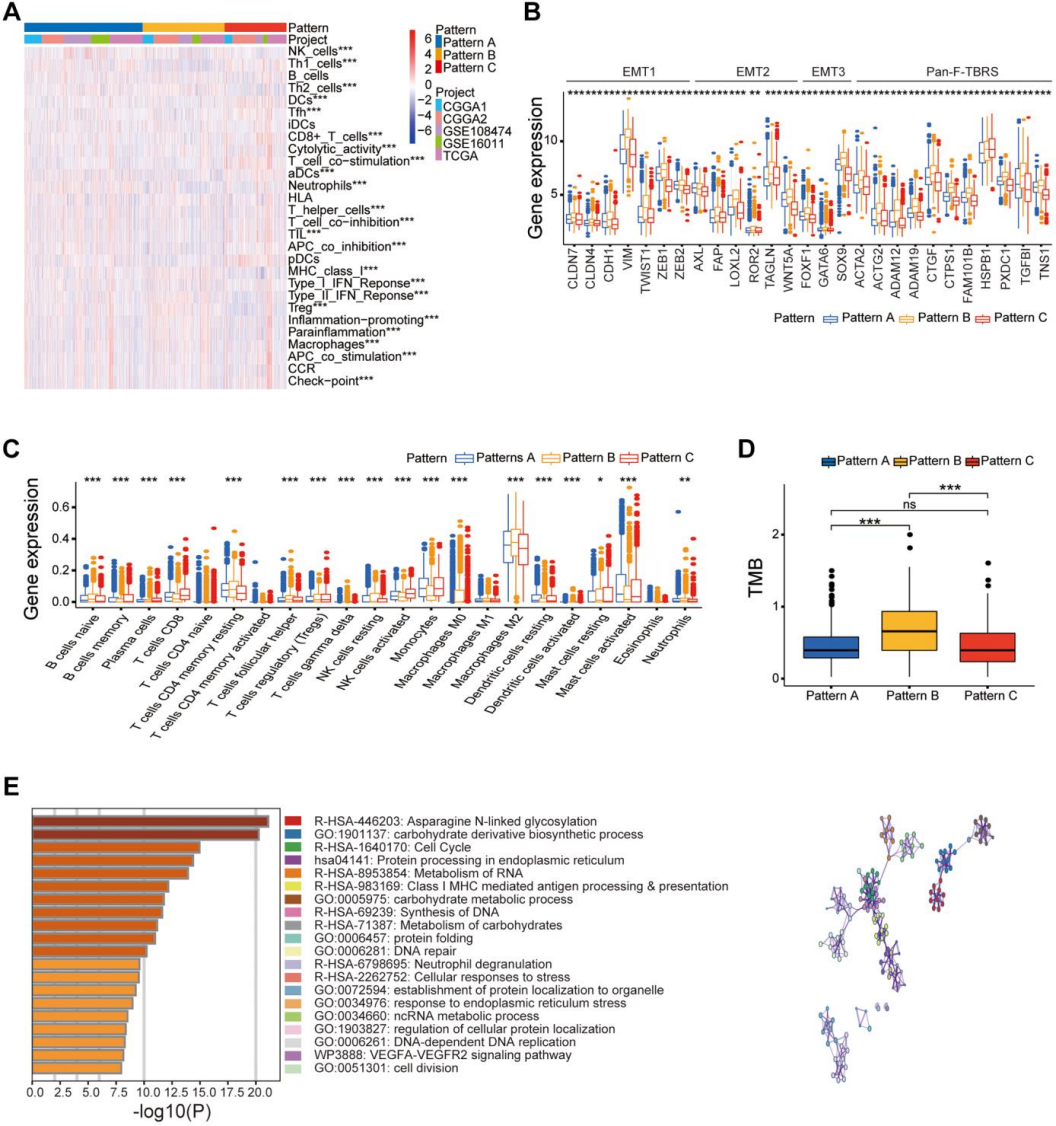
**Different clinical and transcriptome characteristics of the three DNA methylation regulator patterns in the gather glioma cohort.** (**A**) Heatmap of several immune signatures for the three DNA methylation regulator patterns in the gather glioma cohort. (**B**) Box-plots show the expression levels of few stroma-activated related genes in the three DNA methylation regulator patterns based on the gather glioma cohort. (**C**) Box-plots show the proportions of several immune cells types in the three patterns based on the gather glioma cohort. (**D**) Box-plots show the TMB for the three patterns in the TCGA dataset. (**E**) Functional annotation of the DNA methylation related genes between three patterns in the gather glioma cohort. Note: ns *P* > 0.05; ^*^P < 0.05; ^**^P < 0.01; ^***^P < 0.001.

Stromal activation is associated with inhibition of immune cell infiltration and poor prognosis of cancer patients. Therefore, we analyzed the correlation between the three DNA infiltration and several immune cell infiltration and stromal activation signatures. Gliomas with pattern C were significantly rich in immune cell types such as activated DCs, activated NK cells, monocytes, DCs, activated mast cells, co-stimulation T cell, CD8+ T cells, and Tfh (Figure 3A, 3C). These results were in accordance with favorable prognosis of glioma patients with pattern C. Gliomas with patterns B showed significant stromal activation including upregulation of epithelial-mesenchymal transition (EMT)-related genes and pan-fibroblast-THF-β response signature (pan-F-TBRS; Figure 3B). Moreover, pattern B also showed positive correlation with TMB (Figure 3D).

These results showed that all three DNA methylation regulator patterns were associated with significantly distinct immune cell infiltration phenotypes. Pattern A demonstrated immune-excluded tumor phenotype characterized by infiltration of innate immune cell types and stromal activation. Pattern B demonstrated immune-desert tumor phenotype characterized by low immune cell infiltration and immune repression status. Pattern C demonstrated inflamed immune phenotype characterized by high immune cell infiltration into the tumor microenvironment and activation of the adaptive immune response.

### Validation analysis of the three DNA methylation regulator patterns in the TCGA glioma dataset

Next, we classified the patients in the TCGA glioma dataset (*n* = 649) into three clusters based on the expression of the 20 DNA methylation regulators using the package of ConsensusClusterPlus (Supplementary Figure 5A, 5B, 5D). The results for the TCGA dataset were similar to the entire glioma cohort in this study (Supplementary Figure 5C). PCA demonstrated that the 20 DNA methylation regulators formed three distinct patterns (A, B, and C) in the TCGA dataset (Supplementary Figure 5E). Kaplan-Meier survival analysis showed that OS was significantly lower for glioma patients in pattern C compared to those with patterns A and B (*P* < 0.001; Supplementary Figure 5G), probably due to high expression of most DNA methylation regulator in pattern C (Supplementary Figure 5C). Furthermore, glioma in pattern B showed significantly higher proportions of various immune cells types such as activated DCs, CD8+ T cells, activated mast cells, monocytes, activated NK cells, and plasma cells, which was in accordance with significantly increased OS of glioma patients with pattern B (Supplementary Figure 5F). Moreover, higher stromal activity was observed in pattern C including activation of pan-F-TBRS and EMT-related markers (Supplementary Figure 5H).

This integrated analysis demonstrated that glioma patients could be sorted into immune-inflamed, immune-excluded, and immune-desert phenotypes based on the expression levels of 20 DNA methylation regulators both in the gathered glioma cohort and the TCGA glioma dataset.

### Characterization of differences in the clinicopathological parameters and gene expression patterns in the three DNA methylation related gene clusters and estimation of DNA methylation score (DMS)

**Figure 4:**
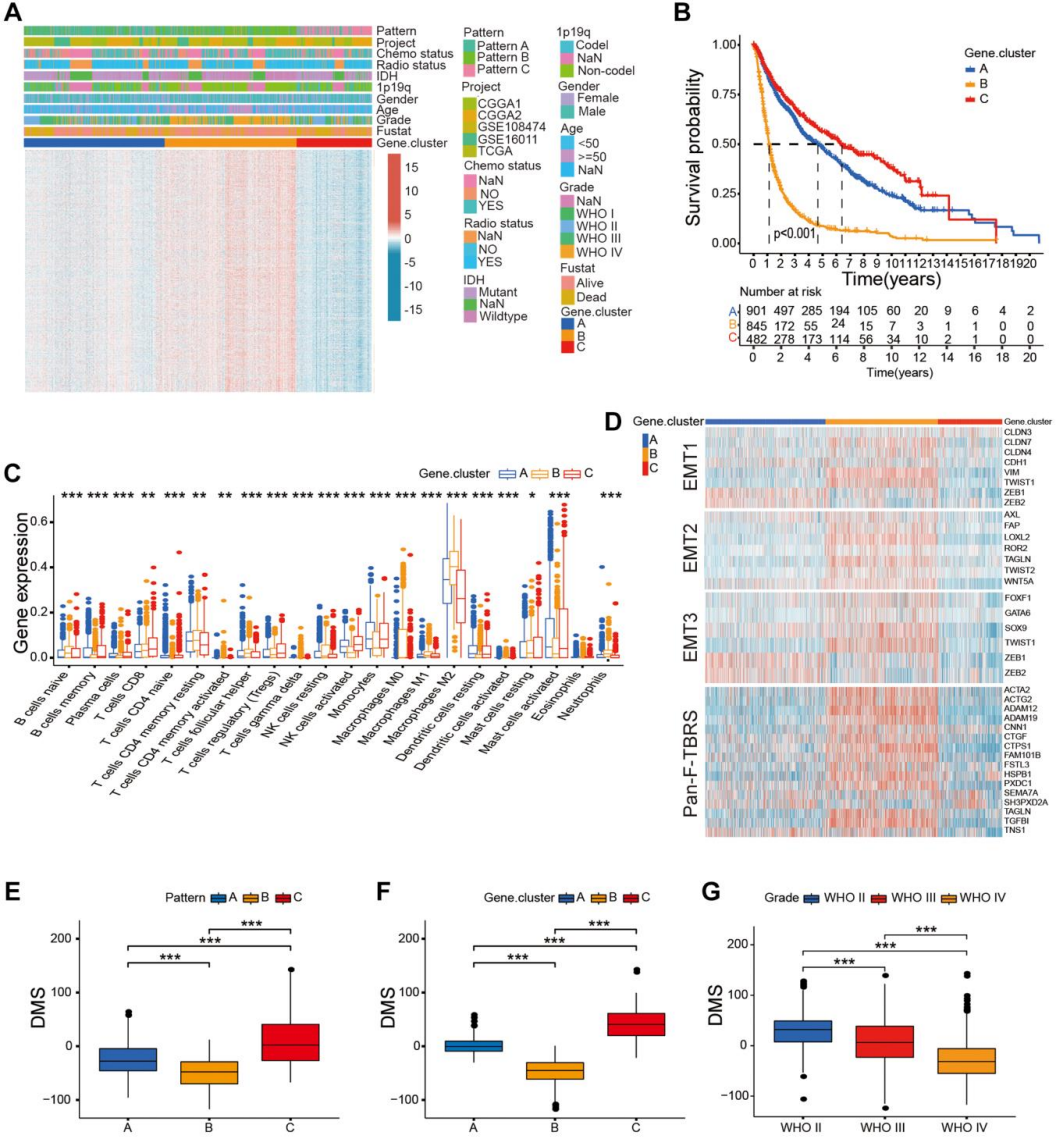
**Construction of DMS in the gather glioma cohort.** (**A**) Unsupervised clustering of the overlapping DNA methylation-related genes in the gather glioma cohorts. (**B**) Survival analysis of glioma patients belonging to the three DNA methylation-related gene clusters; *P* < 0.001. (**C**) The proportion of immune cell types in the glioma and the transcriptome traits in the three DNA methylation-related gene clusters. (**D**) The differences in the expression of genes related to the activated stromal pathways including EMT1, EMT2, EMT3, and pan-F-TBRS between three DNA methylation-related gene clusters. (**E**–**G**) Box-plots shows the DMS for DNA methylation regulator patterns (**E**), gene clusters (**F**), different glioma grades groups (**G**), *P* < 0.001. Note: ns *P* > 0.05; ^*^P < 0.05; ^**^P < 0.01; ^***^P < 0.001.

We then analyzed the DEGs between the glioma belonging to the three distinct patterns of DNA methylation regulator patterns using the “limma” R package and identified 8291 pattern-related DEGs (Supplementary Figure 6D). GO analysis showed that these DEGs were enriched in some immune response and DNA methylation-related pathways such as asparagine N-linked glycosylation, cell cycle, DNA-dependent DNA replication, and Class I MHC-mediated antigen processing and presentation (Figure 3E). Moreover, univariate Cox regression analysis demonstrated that 5679 DEGs were associated with prognosis of the glioma patients. We then classified the glioma patients into different genomic phenotypes based on the expression levels of the 5679 prognosis-related DEGs. Furthermore, we performed unsupervised cluster analysis of the glioma patients based on the expression patterns of 5679 prognosis-related DEGs and identified three distinct gene clusters, A, B, and C (Supplementary Figure 6A–6C). These results confirmed three distinct DNA methylation-related gene clusters in the gliomas. Patients in the DNA methylation-related gene cluster B were associated with WHO IV grade, old age, non-codeletion status of 1p/19q, and wild-type IDH gene status (Figure 4A). Kaplan-Meier survival curves analysis showed that glioma patients in the DNA methylation-related gene cluster B were associated with poorer OS compared to patients in gene clusters A and C (*P* < 0.05; Figure 4B). Moreover, consistent with the clinicopathological features, glioma samples in gene cluster B showed higher proportions of resting CD4 memory T cells, resting NK cells, and resting mast cells in the TME and were significant positively correlated with the activation of EMT and pan-F-TBRS, thereby demonstrating the immune-desert phenotype (Figure 4C, 4D). Glioma in DNA methylation-related gene cluster C showed higher proportions of activated NK cells, activated DCs, and activated mast cells, and were significantly associated with activation of immune-response and immune-checkpoint related genes, thereby demonstrating immune-inflamed phenotype and activated tumor immune microenvironment (Figure 4C; Supplementary Figure 6E).

**Figure 5:**
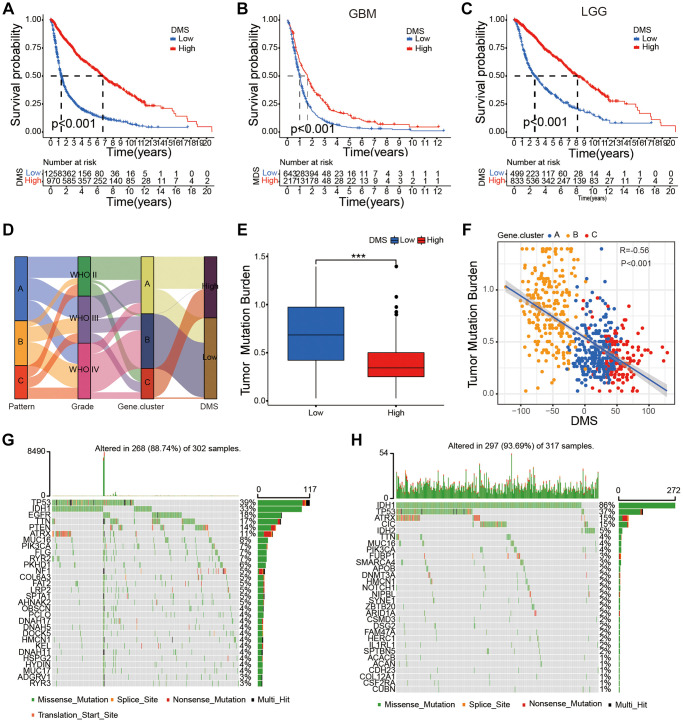
**Survival characteristics of glioma patients based on DMS and the relationship between DMS and tumor somatic mutation.** (**A**–**C**) Survival analyses with the OS rates for the low DMS and high DMS groups among all glioma (**A**), GBM (**B**) and LGG (**C**) samples patients based on gather glioma cohorts, *P* < 0.001. (**D**) Sankey diagram shows the association between DNA methylation regulator patterns, glioma grades, DNA methylation-related gene cluster, and DMS groups. (**E**) Differences in DMS between patients with high and low TMB, *P* < 0.001. (**F**) Scatter plot shows the relationship between DMS and TMB in the glioma samples (R = 0.56; *P* < 0.001). (**G**–**H**) Waterfall plot shows tumor somatic mutations in high (**G**) and low (**H**) DMS subgroups. Note: ns *P* > 0.05; ^*^P < 0.05; ^**^P < 0.01; ^***^P < 0.001.

These results demonstrated that DNA methylation changes contributed to three distinct immune phenotypes in the glioma. However, we could not correctly forecast the DNA methylation status of individual glioma samples using unsupervised cluster analysis. Therefore, we calculated the DNA methylation score (DMS) of each glioma, based on the expression of 5679 prognosis-related DEGs. Our results showed significant difference in the DMS between the three DNA methylation modification patterns and DNA methylation-related gene clusters, with pattern B and gene cluster B showing the lowest median DMS (Figure 4E, 4F). WHO grade II gliomas showed the highest median DMS and WHO grade IV glioma showed the lowest median DMS (Figure 4G). The association between the DNA methylation modification patterns, tumor grade, DNA methylation-related gene clusters, and DMS categories are presented in the Sankey diagram (Figure 5D, Supplementary Table 4).

The glioma samples were classified into low- and high-DMS categories based on a cutoff value of 7.705903. Kaplan-Meier survival analyses showed that OS was significantly shorter in glioma patients with low-DMS compared to those with high-DMS across all grades in the gathered glioma cohort (Figure 5A). Moreover, LGG and GBM patients with low DMS showed significantly shorted OS compared to those with high DMS (*P* < 0.001; Figure 5B, 5C). Further, we analyzed the correlation between DMS and clinicopathological characteristics of the glioma patients. We observed significant differences in the DMS between glioma patient groups stratified by WHO grade (*P* < 0.001), survival status (*P* < 0.001), age (*P* < 0.05), 1p/19q status (*P* < 0.001), and isocitrate dehydrogenase (IDH) status (*P* < 0.01; Supplementary Figure 7A).

Univariate Cox regression analysis showed that WHO grade, age, gender, IDH status, 1p/19q status, and DMS were strongly associated with the OS of glioma patients in our study. Multivariate Cox regression analysis revealed that WHO grade, age, IDH status, 1p/19q status, and DMS were positively correlated with the OS of patients with gliomas (Supplementary Figure 7C). These results confirmed that DMS was an independent prognostic marker in glioma.

### Characterization of DMS in the TCGA dataset and its association with tumor somatic mutations in glioma samples

Next, we further validated the prognostic value of DMS in the glioma samples using the TCGA dataset. Unsupervised cluster analysis showed 5312 DEGs between the three DNA methylation modification patterns and different DNA methylation-related gene clusters (Supplementary Figure 8A–8D). Furthermore, Kaplan-Meier survival curves showed that OS was significantly lower in the glioma patients belonging to DNA methylation gene cluster C compared to those from DNA methylation-related gene clusters A and B (Supplementary Figure 8F); cluster C glioma samples showed high expression levels of most DNA methylation regulatory genes (Supplementary Figure 8E) and positive correlation with activated EMT 1-3 and pan-F-TBRS (Supplementary Figure 8G), thereby representing the immune-desert phenotype.

We classified glioma samples in the TCGA dataset into low- and high-DMS categories using the cutoff value of −28.66371. Kaplan-Meier survival curve showed that OS was significantly shorter for glioma patients with low DMS compared to those with high DMS across different grades based on the TCGA dataset (*P* < 0.001; Supplementary Figure 9A–9C). This data was in agreement with the results for the entire glioma cohort. Our results also validated that DMS was an independent prognostic biomarker for predicting OS of glioma patients in the TCGA cohort (Supplementary Figure 7D). Furthermore, the high DMS group showed significantly OS in glioma patients belonging to different grades (ALL, LGG, and GBM) in the CGGA1 (*P* < 0.001; Supplementary Figure 9D–9F), CGGA2 (*P* < 0.001; Supplementary Figure 9G–9I), GSE180474 (*P* < 0.001; Supplementary Figure 9J–9L), and GSE16011 (*P* < 0.001; Supplementary Figure 9M–9O) datasets. We also observed positive correlation between DMS and other clinicopathological characteristics of the glioma patients based on the TCGA dataset (*P* < 0.01; Supplementary Figure 7B).

We then investigated differences in the TMB between the low DMS and high DMS subgroups of the TCGA glioma dataset. Low-DMS subgroup demonstrated more extensive TMB than the high DMS subgroup (Figure 5G, 5H). TMB quantification analysis showed that gliomas with low DMS were positively associated with higher TMB (Figure 5E, 5F). Our results also showed that gliomas with high DMS positively correlated with high TMB. Previous studies have shown that TMB was negatively associated with OS of glioma patients and high TMB was associated with reduced infiltration of immune cells into the glioma [25, 26]. Therefore, our results would provide novel perspective for exploring the mechanisms of DNA methylation modification in TMB, and shaping of TME landing.

### Predictive value of DMS in immunotherapy

**Figure 6:**
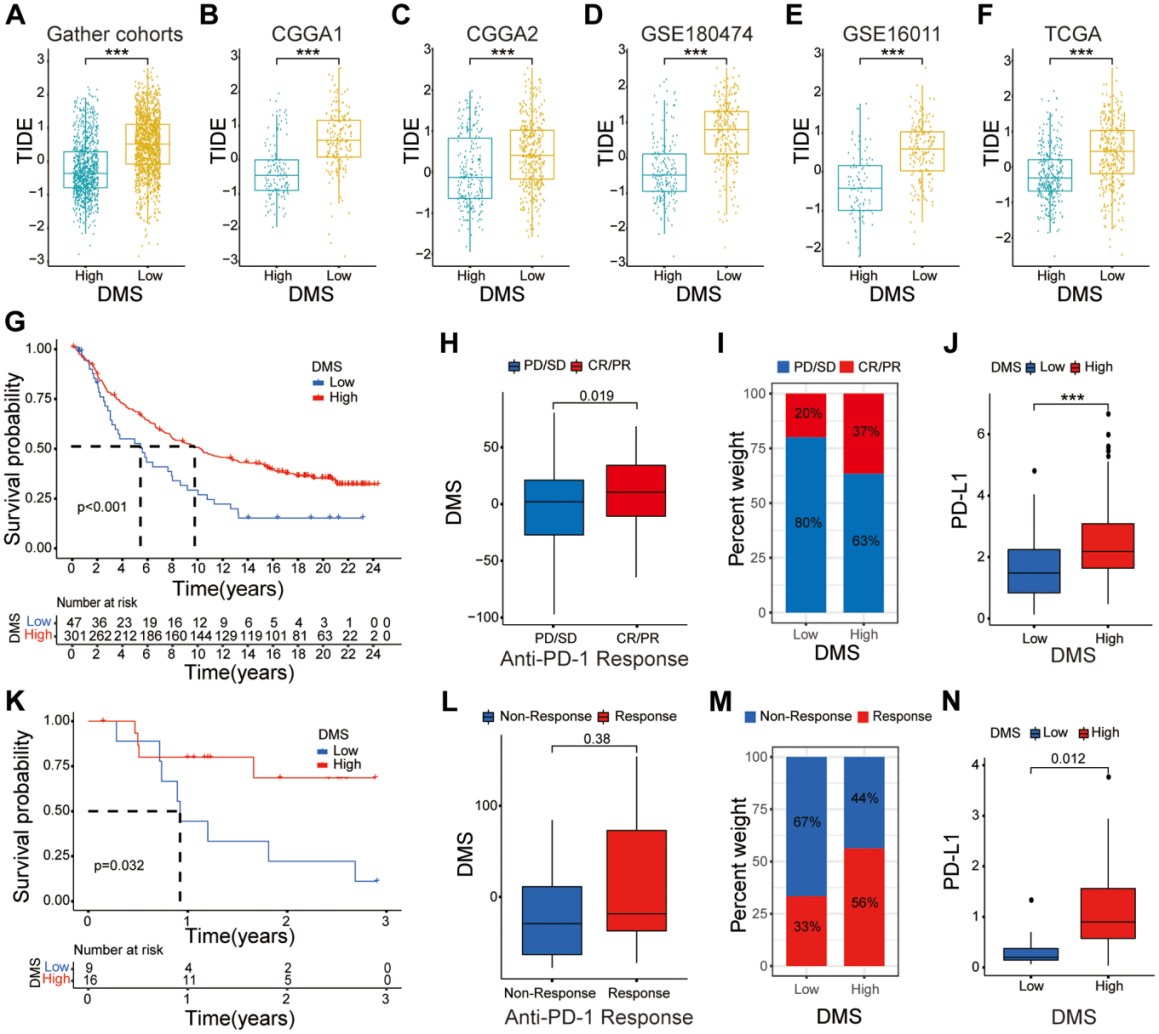
**The relationship between DMS and response to anti-PD-1/L1 immunotherapy.** (**A**–**F**) Distribution of TIDE scores between high and low DMS subgroups in the gather glioma cohorts, as well as CGGA1, CGGA2, GSE180474, GSE16011 and TCGA datasets, respectively. (**G**–**I**) Survival analyses with the OS (**G**), clinical response to anti PD-1 immunotherapy (**H**), proportion of patients responding to PD-1 blockade immunotherapy (**I**), differences in PD-L1 expression (**J**) based on the low and high DMS subgroups in the IMvigor210 cohort. (**K**–**N**) Survival analyses with the OS (**K**), clinical response to anti PD-1 immunotherapy (**L**), proportion of patients responding to PD-1 blockade immunotherapy (**M**), differences in PD-L1 expression (**N**) based on the low and high DMS subgroups in the GSE78220 cohort. Note: ns *P* > 0.05; ^*^P < 0.05; ^**^P < 0.01; ^***^P < 0.001.

CTLA-4 and PD-1 blockades therapies significantly improve survival rates in many cancer types [27, 28]. Tumor mutation load (TML), PD-L1 expression levels, microsatellite instability (MSI) status, Tumor Immune Dysfunction and Exclusion (TIDE) scores, and IPS, are used to determine the status of tumor immune response [29, 30]. Because our study demonstrated strong association between DMS and tumor immune response, we further investigated the correlation between TIDE scores and DMS. We observed significantly reduced TIDE scores in the high-DMS subgroup of the gather glioma cohort and five glioma datasets (Figure 6A–6F). These results demonstrated that the expression levels of DNA methylation regulator significantly influenced the tumor immune response in glioma.

Therefore, we investigated whether DMS affected the clinical response to anti-PD-L1/PD-1 immunotherapy in the IMvigor210 and GSE78220 cohorts. The results showed that tumors with high DMS were associated with significantly increased OS in both immunotherapy cohorts (*P* < 0.05; Figure 6G, 6K). We further demonstrated significant differences in the clinical response of the high DMS subgroup patients to anti-PD-1/PD-L1 immunotherapy compared to the low DMS subgroup (Figure 6H, 6I, 6L, 6M). Furthermore, patients in the high DMS subgroup were positively associated with significantly high expression of PD-L1 in the two immunotherapy cohorts, thereby demonstrating the potential molecular mechanism underling the clinical response to immunotherapy (*P* < 0.05; Figure 6J, 6N).

In summary, our study showed that DNA methylation modification patterns were significantly associated with the immune response of the glioma. Moreover, the DNA methylation-related gene signature accurately predicted clinical responses to anti-PD-1/PD-L1 immunotherapy.

## DISCUSSION

Several studies have reported DNA methylation plays a crucial role in inflammation, innate immunity, and response to immunotherapy through the interplay between 20 DNA methylation regulatory proteins [31, 32]. Most studies have explored the role of individual DNA methylation regulatory genes or the TME in cancer progression [33, 34]. However, systematic analysis of the overall landscape of DNA methylation regulatory genes and their influence on immune cell infiltration into the glioma has not been reported. Our study demonstrated three distinct patterns of DNA methylation regulatory gene expression that correlated with the infiltration and activation status of immune cell types in the glioma. Thus, our findings shed further light into regulatory mechanisms underlying tumor immune infiltration, which significantly affects response to immunotherapies. Therefore, our results may be relevant to future discovery of novel immune inhibitor immunotherapies.

In our study, we identified three DNA methylation regulatory gene expression patterns (patterns A, B, and C) in the entire cohort of glioma patients and the TCGA glioma dataset based on the expression levels of the 20 DNA methylation regulatory genes. Moreover, these three DNA methylation regulatory gene expression patterns correlated with three distinct tumor immune response phenotypes, namely, immune-excluded for pattern A, immune-desert for pattern B, immune-inflamed phenotype for pattern C. Glioma patients belonging to the DNA methylation regulatory gene expression pattern C were significantly enriched with immune cell types such an activated DCs, CD8+ T cells, co-stimulatory T cells, activated mast cells, and activated NK cells. They were also significantly enriched in immune-related pathways, such as the chemokine signaling pathway and cytokine-cytokine receptor interactions. This suggested that pattern C represented an active tumor immune microenvironment with activated adaptive immune system, which also correlated with better prognosis. In contrast to pattern C, pattern B was associated with poor prognosis and showed significantly higher stromal activity including activation of EMT and pan-F-TBRS, thereby suggesting presence of a cold or inactive tumor immune microenvironment. Although pattern A was markedly enriched in immune-related pathways, it was classified as immune-excluded phenotype because it was characterized by innate immune cell infiltration and stromal activation.

We identified 8291 DEGs by comparing the three distinct DNA methylation regulatory gene expression patterns. The expression of these DEGs correlated with the status of DNA methylation and immune-related pathways. Furthermore, we identified the three DNA methylation-related gene clusters based on the expression of prognosis-related DEGs, which were associated with immune or stromal activation. This confirmed three distinct immune subtypes in the gliomas.

We then developed a risk score system (DMS) to identify and quantify heterogeneity in the DNA methylation modifications between various glioma samples. The immune-inflamed subtype and WHO II subgroup showed the highest median DMS. In addition, DMS was associated with several clinicopathological characteristics of glioma samples such as WHO grade, age, 1p/19q co-deletion status, survival status, and IDH status. Multivariate Cox regression analysis confirmed that DMS was an independent prognostic biomarker for evaluating OS of glioma patients.

Our data also showed that glioma patients with low DMS were associated with higher TMB, and positively correlated with expression of EMT and pan-F-TBRS. Previous studies have shown that stromal activation correlates with resistance to immunotherapy [35, 36]. This suggested that in addition to antigen processing and improved cytolytic activity, efficacy of immunotherapy was associated with suppression of angiogenesis and activation of EMT related pathway, which contributed to reduce infiltration of T cells into the tumors and effective killing of cancer cells. Several studies showed that TME played a significant role in immunotherapeutic efficacy [37–39]. Moreover, the levels of activated T cells, activated macrophages, activated DCs, and activated NK cells were associated with efficacy of immune responses [40–42].

Distinct stromal activation and immune infiltration landscape of the three patterns suggested DMS correlated with clinical responses to immune checkpoint blockade therapy. Furthermore, our study showed that DMS was an independent predictive biomarker for immunotherapy outcomes, in addition to other well-established biomarkers such as TME, neoantigen load, PD-L1 expression, stromal and immune status, and TIDE. This implied that integration of DMS with other predictive biomarker may provide more effective strategy for immunotherapy. DMS also showed good predictive value in the two independent cohorts of cancer patients that underwent anti-PD-1/PD-L1 immunotherapy. Thus, our study suggested that the DNA methylation regulator patterns regulated tumor immune response phenotypes and might guide therapeutic strategies.

In conclusion, we systematically analyzed the expression levels of 20 DNA methylation regulatory genes in 2228 glioma patients and identified three DNA methylation modification patterns. We demonstrated significant association between the three DNA methylation modification patterns with the immune cell infiltration status TME of the glioma tissues. Furthermore, we estimated DMS of individual glioma samples based on the expression levels of DNA methylation-related DEGs and identified three distinct immune phenotypes that could guide therapeutic strategies and immunotherapeutic responses. We also demonstrated that DMS was an independent biomarker for predicting prognosis of glioma patients.

## MATERIALS AND METHODS

### Pre-processing of patient data from multiple glioma datasets and two immune-checkpoint blockade cohorts

The flow chart of our research strategy is shown in Supplementary Figure 1A. We evaluated mRNA expression data and clinicopathological information of glioma patients in The Cancer Genome Atlas (TCGA), GEO, and Chinese Glioma Genome Atlas (CGGA) databases. We excluded samples with missing prognostic information. Finally, five glioma datasets including 2228 glioma samples ((TCGA (*n* = 648), CGGA1 (*n* = 309), CGGA2 (*n* = 593), and GEO: GSE16011 (*n* = 263), GSE108474 (*n* = 415)) were included for further analysis. The mRNA expression and clinicopathological information for the TCGA glioma datasets were downloaded from the University of California Santa Cruz (UCSC) Xena browser. The mRNA expression and clinicopathological information for the glioma samples in the GSE16011 and GSE108474 datasets were downloaded from the GEO database (https://www.ncbi.nlm.nih.gov/geo/query/acc.cgi?) and those from the CGGA1 and CGGA2 datasets were downloaded from CGGA database (http://www.cgga.org.cn/). We also downloaded mRNA expression and clinicopathological data of patients in the two independent anti-PD-L1 immunotherapy cohorts, namely, IMvigor210, which included 298 urothelial cancer patients that underwent atezolizumab treatment (*n* = 298; http://research-pub.gene.com/IMvigor210CoreBiologies/); and GSE78220, included 26 metastatic melanoma patients that underwent treatment with pembrolizumab (*n* = 26; https://www.ncbi.nlm.nih.gov/geo/query/acc.cgi?) [11, 43]. Then the gene expression data of glioma samples was transformed into transcripts per kilobase million (TPM) values, and we merged and removed batch effect from non-biological technical biases by the “ComBat” algorithm of sva package. The information of the five gathered glioma databases was shown in Supplementary Table 1. The genomic mutation data was obtained from the UCSC Xena browse. The RNA-seq data of all samples was normalized with the following formula: log_2_ (N+1).

### Use of immunohistochemistry assay to validate bioinformatics results

We collected 10 NBTs and 10 LGG tissues from the Second Affiliated Hospital of Nanchang University from June 2020 to April 2022. Our study was approved by the Ethics Committee of this hospital. We performed the immunohistochemistry assay on human tissues by methods described preciously.

### Protein-protein interaction network between 20 DNA methylation regulators

We retrieved the literatures databases and identified 20 DNA methylation regulatory genes for analysis in this study, containing 3 writers, 3 erasers, and 14 readers. The STRING and Cytoscape databases were used to construct the protein-protein interaction (PPI) network between these 20 DNA methylation regulatory proteins [44, 45].

### Unsupervised clustering of DNA methylation regulatory genes

The samples in the five integrated glioma datasets were classified according to distinct DNA methylation modification patterns based on the expression levels of various DNA methylation regulatory genes using the R package “ConsensusClusterPlus” [46]. The Euclidean distance was used to calculate similarity distance between the glioma patients. The k-means algorithm was used for validation with 50 iterations and 1000 times repetitions. Principal component analysis (PCA) was performed to verify the classification into multiple patterns based on DNA methylation regulatory genes expression.

### Gene set variation analysis and annotation of gene ontology terms

The gene set variation analysis (GSVA) R package was used to identify molecular functions related to different patterns of DNA methylation modifications. The gene signatures were obtained from the Molecular Signature Database (MSigDB) using the gene set “c2.cp.kegg.v6.2 symbols” [47]. The differentially expressed genes (DEGs) between the different patterns of DNA methylation modifications were obtained using the R package of “limma” [48]. Geno Ontology (GO) analysis was performed to determine the biological functions related to the DNA methylation regulatory related genes using *P* < 0.05 as the cut-off value [49].

### Estimation of the relative abundance of immune cell types in the TME of gliomas

The single-sample gene-set enrichment analysis (ssGSEA) formula was performed using the “GSVA” R package to determine the relative abundance of the immune cell types in the glioma TME. A list of representative marker genes that represent different immune cells types were acquired from Charoentong’s study (Supplementary Table 5) that summarized 366 microarrays of several immune cell types containing activated mast cells, plasma cells, activated dendritic cells (DCs), activated NK cells, natural killer T cells, activated CD8+ T cells, eosinophils, activated CD4+ T cells, macrophages, and others [50, 51]. The enrichment scores were computed using the ssGSEA algorithm to determine the relative abundance of tumor-infiltrating immune cells in the glioma patients.

### Generation of the DNA methylation score (DMS)

We developed a risk score system based on the DNA methylation modification in the glioma samples. The DNA methylation score (DMS) was calculated by: first identifying survival-associated DEGs using the “survival” R package, PCA was used to evaluate DMS by selecting PC1 and PC2, DMS was based on the largest block of highly correlated survival-associated DEGs and was calculated as follows: DMS = ∑ (PC1_i_ + PC2_i_), where i, represents the expression of survival-associated DEGs expression in the cohort of glioma patients [52, 53].

### Statistical analysis

The Shapiro-Wilk normality test was used to analyze normality of the variables [54]. The expression levels of DNA methylation regulators between two normally distributed groups were analyzed using unpaired *t* tests, whereas, the Wilcoxon rank-sum test was used to analyze non-normally distributed variables. Kruskal-Wallis or one-way ANOVA tests were used to compare differential gene expression between the three subgroups [55]. Spearman and distance correlation analyses were performed to determine the relationships between different subgroups. We computed the best cutoff point for the overall survival (OS) rates using R package of “survminer”. Then prognostic analysis was performed using the Kaplan-Meier survival curves [56]. Log-rank tests were used to estimate the differences between pairs of glioma subtypes. Univariate and multivariate Cox regression analysis was performed to determine the hazard ratios (HRs) for the 20 DNA methylation regulators and to determine if DMS was an independent prognostic biomarker. The mutation landscape of the DNA methylation regulatory genes in the TCGA dataset was determined using “maftools” R package, the landscape of copy number variation (CNVs) in the DNA methylation regulatory genes was determined using the “RCircos” R package [57]. Statistical analysis was performed using the R programming language v3.6.3, *P* < 0.05 was considered statistically significant.

## Supplementary Materials

Supplementary Figures

Supplementary Table 1

Supplementary Table 2

Supplementary Table 3

Supplementary Table 4

Supplementary Table 5

## References

[1] Schübeler D. Function and information content of DNA methylation. Nature. 2015; 517:321–6. 10.1038/nature14192 25592537

[2] Koch A, Joosten SC, Feng Z, de Ruijter TC, Draht MX, Melotte V, Smits KM, Veeck J, Herman JG, Van Neste L, Van Criekinge W, De Meyer T, van Engeland M. Analysis of DNA methylation in cancer: location revisited. Nat Rev Clin Oncol. 2018; 15:459–66. 10.1038/s41571-018-0004-4 29666440

[3] Horvath S, Raj K. DNA methylation-based biomarkers and the epigenetic clock theory of ageing. Nat Rev Genet. 2018; 19:371–84. 10.1038/s41576-018-0004-3 29643443

[4] Capper D, Jones DTW, Sill M, Hovestadt V, Schrimpf D, Sturm D, Koelsche C, Sahm F, Chavez L, Reuss DE, Kratz A, Wefers AK, Huang K, et al. DNA methylation-based classification of central nervous system tumours. Nature. 2018; 555:469–74. 10.1038/nature26000 29539639 PMC6093218

[5] Cloughesy TF, Petrecca K, Walbert T, Butowski N, Salacz M, Perry J, Damek D, Bota D, Bettegowda C, Zhu JJ, Iwamoto F, Placantonakis D, Kim L, et al. Effect of Vocimagene Amiretrorepvec in Combination With Flucytosine vs Standard of Care on Survival Following Tumor Resection in Patients With Recurrent High-Grade Glioma: A Randomized Clinical Trial. JAMA Oncol. 2020; 6:1939–46. 10.1001/jamaoncol.2020.3161 33119048 PMC7596685

[6] Wang DD, Deng H, Hervey-Jumper SL, Molinaro AA, Chang EF, Berger MS. Seizure Outcome After Surgical Resection of Insular Glioma. Neurosurgery. 2018; 83:709–18. 10.1093/neuros/nyx486 29126238 PMC6454798

[7] Sollmann N, Gutbrod-Fernandez M, Burian E, Riederer I, Meyer B, Hock A, Gempt J, Zimmer C, Kirschke JS. Subtraction Maps Derived from Longitudinal Magnetic Resonance Imaging in Patients with Glioma Facilitate Early Detection of Tumor Progression. Cancers (Basel). 2020; 12:3111. 10.3390/cancers12113111 33114383 PMC7692500

[8] Jung E, Osswald M, Ratliff M, Dogan H, Xie R, Weil S, Hoffmann DC, Kurz FT, Kessler T, Heiland S, von Deimling A, Sahm F, Wick W, Winkler F. Tumor cell plasticity, heterogeneity, and resistance in crucial microenvironmental niches in glioma. Nat Commun. 2021; 12:1014. 10.1038/s41467-021-21117-3 33579922 PMC7881116

[9] Deland K, Starr BF, Mercer JS, Byemerwa J, Crabtree DM, Williams NT, Luo L, Ma Y, Chen M, Becher OJ, Kirsch DG. Tumor genotype dictates radiosensitization after Atm deletion in primary brainstem glioma models. J Clin Invest. 2021; 131:142158. 10.1172/JCI142158 32990677 PMC7773366

[10] Rizvi NA, Hellmann MD, Snyder A, Kvistborg P, Makarov V, Havel JJ, Lee W, Yuan J, Wong P, Ho TS, Miller ML, Rekhtman N, Moreira AL, et al. Cancer immunology. Mutational landscape determines sensitivity to PD-1 blockade in non-small cell lung cancer. Science. 2015; 348:124–8. 10.1126/science.aaa1348 25765070 PMC4993154

[11] Hugo W, Zaretsky JM, Sun L, Song C, Moreno BH, Hu-Lieskovan S, Berent-Maoz B, Pang J, Chmielowski B, Cherry G, Seja E, Lomeli S, Kong X, et al. Genomic and Transcriptomic Features of Response to Anti-PD-1 Therapy in Metastatic Melanoma. Cell. 2017; 168:542. 10.1016/j.cell.2017.01.010 28129544

[12] Ferris RL, Blumenschein G Jr, Fayette J, Guigay J, Colevas AD, Licitra L, Harrington K, Kasper S, Vokes EE, Even C, Worden F, Saba NF, Iglesias Docampo LC, et al. Nivolumab for Recurrent Squamous-Cell Carcinoma of the Head and Neck. N Engl J Med. 2016; 375:1856–67. 10.1056/NEJMoa1602252 27718784 PMC5564292

[13] Gu SS, Zhang W, Wang X, Jiang P, Traugh N, Li Z, Meyer C, Stewig B, Xie Y, Bu X, Manos MP, Font-Tello A, Gjini E, et al. Therapeutically Increasing MHC-I Expression Potentiates Immune Checkpoint Blockade. Cancer Discov. 2021; 11:1524–41. 10.1158/2159-8290.CD-20-0812 33589424 PMC8543117

[14] Kang YK, Boku N, Satoh T, Ryu MH, Chao Y, Kato K, Chung HC, Chen JS, Muro K, Kang WK, Yeh KH, Yoshikawa T, Oh SC, et al. Nivolumab in patients with advanced gastric or gastro-oesophageal junction cancer refractory to, or intolerant of, at least two previous chemotherapy regimens (ONO-4538-12, ATTRACTION-2): a randomised, double-blind, placebo-controlled, phase 3 trial. Lancet. 2017; 390:2461–71. 10.1016/S0140-6736(17)31827-5 28993052

[15] Liu Y, Wang Z, Li X, Ma X, Wang S, Kang F, Yang W, Ma W, Wang J. Near-Infrared Fluorescent Peptides with High Tumor Selectivity: Novel Probes for Image-Guided Surgical Resection of Orthotopic Glioma. Mol Pharm. 2019; 16:108–17. 10.1021/acs.molpharmaceut.8b00888 30517013

[16] Fuchs CS, Doi T, Jang RW, Muro K, Satoh T, Machado M, Sun W, Jalal SI, Shah MA, Metges JP, Garrido M, Golan T, Mandala M, et al. Safety and Efficacy of Pembrolizumab Monotherapy in Patients With Previously Treated Advanced Gastric and Gastroesophageal Junction Cancer: Phase 2 Clinical KEYNOTE-059 Trial. JAMA Oncol. 2018; 4:e180013. 10.1001/jamaoncol.2018.0013 29543932 PMC5885175

[17] Jones PA, Ohtani H, Chakravarthy A, De Carvalho DD. Epigenetic therapy in immune-oncology. Nat Rev Cancer. 2019; 19:151–61. 10.1038/s41568-019-0109-9 30723290

[18] Dunn J, Rao S. Epigenetics and immunotherapy: The current state of play. Mol Immunol. 2017; 87:227–39. 10.1016/j.molimm.2017.04.012 28511092

[19] Phillips RE, Soshnev AA, Allis CD. Epigenomic Reprogramming as a Driver of Malignant Glioma. Cancer Cell. 2020; 38:647–60. 10.1016/j.ccell.2020.08.008 32916125 PMC8248764

[20] Wu HX, Chen YX, Wang ZX, Zhao Q, He MM, Wang YN, Wang F, Xu RH. Alteration in TET1 as potential biomarker for immune checkpoint blockade in multiple cancers. J Immunother Cancer. 2019; 7:264. 10.1186/s40425-019-0737-3 31623662 PMC6798429

[21] Xu YP, Lv L, Liu Y, Smith MD, Li WC, Tan XM, Cheng M, Li Z, Bovino M, Aubé J, Xiong Y. Tumor suppressor TET2 promotes cancer immunity and immunotherapy efficacy. J Clin Invest. 2019; 129:4316–31. 10.1172/JCI129317 31310587 PMC6763236

[22] Moore LD, Le T, Fan G. DNA methylation and its basic function. Neuropsychopharmacology. 2013; 38:23–38. 10.1038/npp.2012.112 22781841 PMC3521964

[23] Tyagi SC, Stanisic D, Singh M. Epigenetic memory: gene writer, eraser and homocysteine. Mol Cell Biochem. 2021; 476:507–12. 10.1007/s11010-020-03895-4 33030620

[24] Cao J, Yan Q. Cancer Epigenetics, Tumor Immunity, and Immunotherapy. Trends Cancer. 2020; 6:580–92. 10.1016/j.trecan.2020.02.003 32610068 PMC7330177

[25] Hodges TR, Ott M, Xiu J, Gatalica Z, Swensen J, Zhou S, Huse JT, de Groot J, Li S, Overwijk WW, Spetzler D, Heimberger AB. Mutational burden, immune checkpoint expression, and mismatch repair in glioma: implications for immune checkpoint immunotherapy. Neuro Oncol. 2017; 19:1047–57. 10.1093/neuonc/nox026 28371827 PMC5570198

[26] Prost D, Bielle F, Ligon KL, Touat M. Mutational burden and immune recognition of gliomas. Curr Opin Oncol. 2021; 33:626–34. 10.1097/CCO.0000000000000787 34651608

[27] Chuntova P, Chow F, Watchmaker PB, Galvez M, Heimberger AB, Newell EW, Diaz A, DePinho RA, Li MO, Wherry EJ, Mitchell D, Terabe M, Wainwright DA, et al. Unique challenges for glioblastoma immunotherapy-discussions across neuro-oncology and non-neuro-oncology experts in cancer immunology. Meeting Report from the 2019 SNO Immuno-Oncology Think Tank. Neuro Oncol. 2021; 23:356–75. 10.1093/neuonc/noaa277 33367885 PMC7992879

[28] Rey-Cárdenas M, Guerrero-Ramos F, Gómez de Liaño Lista A, Carretero-González A, Bote H, Herrera-Juárez M, Carril-Ajuria L, Martín-Soberón M, Sepulveda JM, Billalabeitia EG, Castellano D, de Velasco G. Recent advances in neoadjuvant immunotherapy for urothelial bladder cancer: What to expect in the near future. Cancer Treat Rev. 2021; 93:102142. 10.1016/j.ctrv.2020.102142 33453566

[29] Chen H, Yang M, Wang Q, Song F, Li X, Chen K. The new identified biomarkers determine sensitivity to immune check-point blockade therapies in melanoma. Oncoimmunology. 2019; 8:1608132. 10.1080/2162402X.2019.1608132 31413919 PMC6682357

[30] Chen H, Chong W, Wu Q, Yao Y, Mao M, Wang X. Association of *LRP1B* Mutation With Tumor Mutation Burden and Outcomes in Melanoma and Non-small Cell Lung Cancer Patients Treated With Immune Check-Point Blockades. Front Immunol. 2019; 10:1113. 10.3389/fimmu.2019.01113 31164891 PMC6536574

[31] Chakravarthy A, Furness A, Joshi K, Ghorani E, Ford K, Ward MJ, King EV, Lechner M, Marafioti T, Quezada SA, Thomas GJ, Feber A, Fenton TR. Pan-cancer deconvolution of tumour composition using DNA methylation. Nat Commun. 2018; 9:3220. 10.1038/s41467-018-05570-1 30104673 PMC6089972

[32] Zhang MW, Fujiwara K, Che X, Zheng S, Zheng L. DNA methylation in the tumor microenvironment. J Zhejiang Univ Sci B. 2017; 18:365–72. 10.1631/jzus.B1600579 28471108 PMC5442975

[33] Mitra S, Lauss M, Cabrita R, Choi J, Zhang T, Isaksson K, Olsson H, Ingvar C, Carneiro A, Staaf J, Ringnér M, Nielsen K, Brown KM, Jönsson G. Analysis of DNA methylation patterns in the tumor immune microenvironment of metastatic melanoma. Mol Oncol. 2020; 14:933–50. 10.1002/1878-0261.12663 32147909 PMC7191190

[34] Lu C, Wei Y, Wang X, Zhang Z, Yin J, Li W, Chen L, Lyu X, Shi Z, Yan W, You Y. DNA-methylation-mediated activating of lncRNA SNHG12 promotes temozolomide resistance in glioblastoma. Mol Cancer. 2020; 19:28. 10.1186/s12943-020-1137-5 32039732 PMC7011291

[35] Derynck R, Turley SJ, Akhurst RJ. TGFβ biology in cancer progression and immunotherapy. Nat Rev Clin Oncol. 2021; 18:9–34. 10.1038/s41571-020-0403-1 32710082 PMC9721352

[36] Ren Q, Zhu P, Zhang H, Ye T, Liu D, Gong Z, Xia X. Identification and validation of stromal-tumor microenvironment-based subtypes tightly associated with PD-1/PD-L1 immunotherapy and outcomes in patients with gastric cancer. Cancer Cell Int. 2020; 20:92. 10.1186/s12935-020-01173-3 32226313 PMC7092673

[37] Bader JE, Voss K, Rathmell JC. Targeting Metabolism to Improve the Tumor Microenvironment for Cancer Immunotherapy. Mol Cell. 2020; 78:1019–33. 10.1016/j.molcel.2020.05.034 32559423 PMC7339967

[38] Musetti S, Huang L. Nanoparticle-Mediated Remodeling of the Tumor Microenvironment to Enhance Immunotherapy. ACS Nano. 2018; 12:11740–55. 10.1021/acsnano.8b05893 30508378

[39] Pitt JM, Marabelle A, Eggermont A, Soria JC, Kroemer G, Zitvogel L. Targeting the tumor microenvironment: removing obstruction to anticancer immune responses and immunotherapy. Ann Oncol. 2016; 27:1482–92. 10.1093/annonc/mdw168 27069014

[40] Sabado RL, Balan S, Bhardwaj N. Dendritic cell-based immunotherapy. Cell Res. 2017; 27:74–95. 10.1038/cr.2016.157 28025976 PMC5223236

[41] Waldman AD, Fritz JM, Lenardo MJ. A guide to cancer immunotherapy: from T cell basic science to clinical practice. Nat Rev Immunol. 2020; 20:651–68. 10.1038/s41577-020-0306-5 32433532 PMC7238960

[42] Shimasaki N, Jain A, Campana D. NK cells for cancer immunotherapy. Nat Rev Drug Discov. 2020; 19:200–18. 10.1038/s41573-019-0052-1 31907401

[43] Mariathasan S, Turley SJ, Nickles D, Castiglioni A, Yuen K, Wang Y, Kadel EE III, Koeppen H, Astarita JL, Cubas R, Jhunjhunwala S, Banchereau R, Yang Y, et al. TGFβ attenuates tumour response to PD-L1 blockade by contributing to exclusion of T cells. Nature. 2018; 554:544–8. 10.1038/nature25501 29443960 PMC6028240

[44] Szklarczyk D, Gable AL, Lyon D, Junge A, Wyder S, Huerta-Cepas J, Simonovic M, Doncheva NT, Morris JH, Bork P, Jensen LJ, Mering CV. STRING v11: protein-protein association networks with increased coverage, supporting functional discovery in genome-wide experimental datasets. Nucleic Acids Res. 2019; 47:D607–13. 10.1093/nar/gky1131 30476243 PMC6323986

[45] Shannon P, Markiel A, Ozier O, Baliga NS, Wang JT, Ramage D, Amin N, Schwikowski B, Ideker T. Cytoscape: a software environment for integrated models of biomolecular interaction networks. Genome Res. 2003; 13:2498–504. 10.1101/gr.1239303 14597658 PMC403769

[46] Wilkerson MD, Hayes DN. ConsensusClusterPlus: a class discovery tool with confidence assessments and item tracking. Bioinformatics. 2010; 26:1572–3. 10.1093/bioinformatics/btq170 20427518 PMC2881355

[47] Hänzelmann S, Castelo R, Guinney J. GSVA: gene set variation analysis for microarray and RNA-seq data. BMC Bioinformatics. 2013; 14:7. 10.1186/1471-2105-14-7 23323831 PMC3618321

[48] Ritchie ME, Phipson B, Wu D, Hu Y, Law CW, Shi W, Smyth GK. limma powers differential expression analyses for RNA-sequencing and microarray studies. Nucleic Acids Res. 2015; 43:e47. 10.1093/nar/gkv007 25605792 PMC4402510

[49] Yu G, Wang LG, Han Y, He QY. clusterProfiler: an R package for comparing biological themes among gene clusters. OMICS. 2012; 16:284–7. 10.1089/omi.2011.0118 22455463 PMC3339379

[50] Charoentong P, Finotello F, Angelova M, Mayer C, Efremova M, Rieder D, Hackl H, Trajanoski Z. Pan-cancer Immunogenomic Analyses Reveal Genotype-Immunophenotype Relationships and Predictors of Response to Checkpoint Blockade. Cell Rep. 2017; 18:248–62. 10.1016/j.celrep.2016.12.019 28052254

[51] Barbie DA, Tamayo P, Boehm JS, Kim SY, Moody SE, Dunn IF, Schinzel AC, Sandy P, Meylan E, Scholl C, Fröhling S, Chan EM, Sos ML, et al. Systematic RNA interference reveals that oncogenic KRAS-driven cancers require TBK1. Nature. 2009; 462:108–12. 10.1038/nature08460 19847166 PMC2783335

[52] Zeng D, Li M, Zhou R, Zhang J, Sun H, Shi M, Bin J, Liao Y, Rao J, Liao W. Tumor Microenvironment Characterization in Gastric Cancer Identifies Prognostic and Immunotherapeutically Relevant Gene Signatures. Cancer Immunol Res. 2019; 7:737–50. 10.1158/2326-6066.CIR-18-0436 30842092

[53] Sotiriou C, Wirapati P, Loi S, Harris A, Fox S, Smeds J, Nordgren H, Farmer P, Praz V, Haibe-Kains B, Desmedt C, Larsimont D, Cardoso F, et al. Gene expression profiling in breast cancer: understanding the molecular basis of histologic grade to improve prognosis. J Natl Cancer Inst. 2006; 98:262–72. 10.1093/jnci/djj052 16478745

[54] Ghasemi A, Zahediasl S. Normality tests for statistical analysis: a guide for non-statisticians. Int J Endocrinol Metab. 2012; 10:486–9. 10.5812/ijem.3505 23843808 PMC3693611

[55] Hazra A, Gogtay N. Biostatistics Series Module 3: Comparing Groups: Numerical Variables. Indian J Dermatol. 2016; 61:251–60. 10.4103/0019-5154.182416 27293244 PMC4885176

[56] Guyot P, Ades AE, Ouwens MJ, Welton NJ. Enhanced secondary analysis of survival data: reconstructing the data from published Kaplan-Meier survival curves. BMC Med Res Methodol. 2012; 12:9. 10.1186/1471-2288-12-9 22297116 PMC3313891

[57] Mayakonda A, Lin DC, Assenov Y, Plass C, Koeffler HP. Maftools: efficient and comprehensive analysis of somatic variants in cancer. Genome Res. 2018; 28:1747–56. 10.1101/gr.239244.118 30341162 PMC6211645

