# Supplementary material for: DNA methylation regulator-mediated modification patterns and tumor microenvironment characterization in glioma
Source: Aging (Albany NY). 2022 Sep 21;14(19):7824–50. doi: 10.18632/aging.204291 (PMC9596205; doi:10.18632/aging.204291)
Supplement: Supplementary Figures [file aging-14-204291-s001.pdf]

SUPPLEMENTARY FIGURES

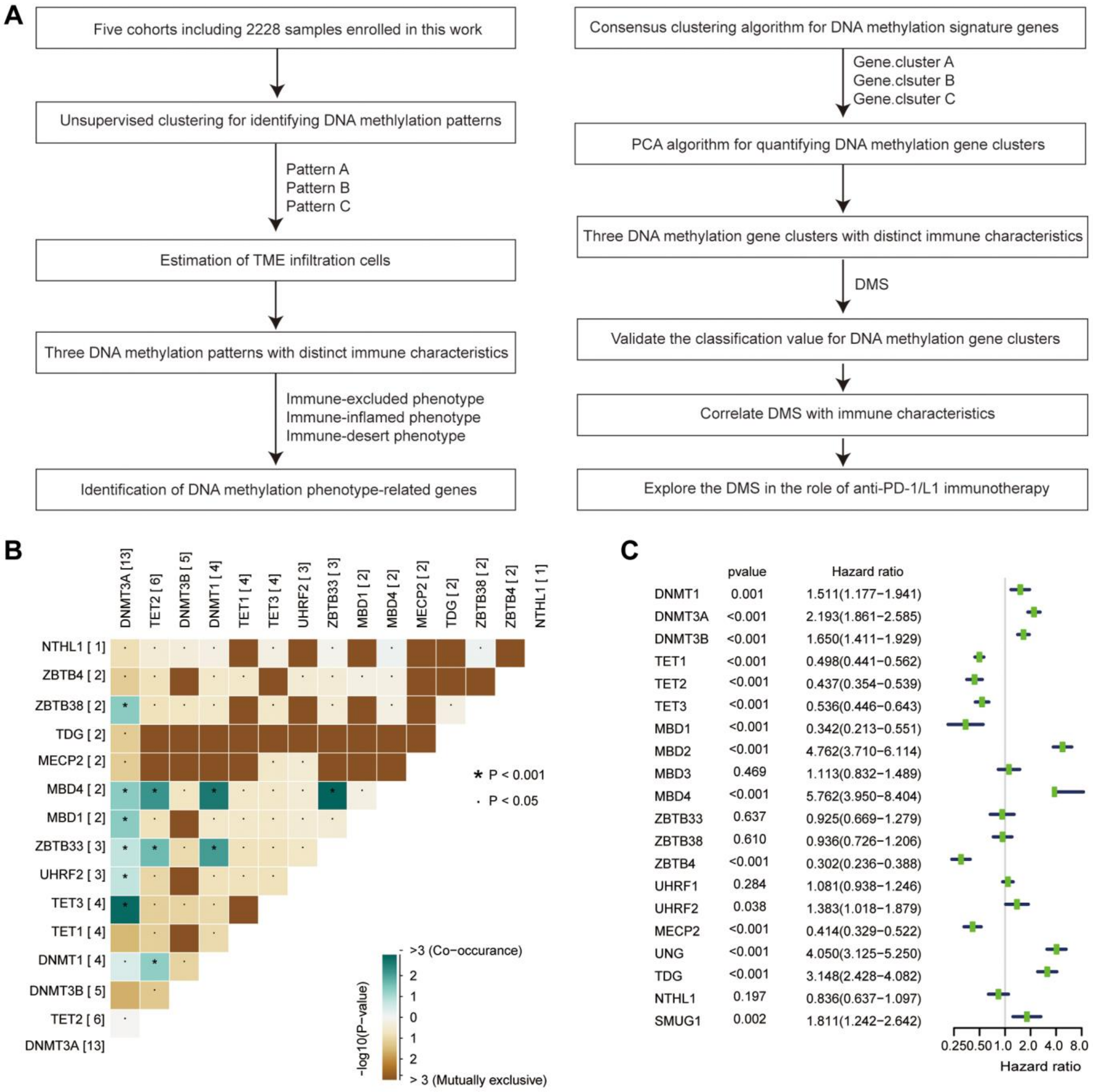

Supplementary Figure 1. Overview of study design and prognostic analysis of 20 DNA methylation regulator. (**A**) Overview of this work. (**B**) The mutation co-occurrence and exclusion analyses for 20 DNA methylation regulator. (**C**) The prognostic analyses for 20 DNA methylation regulator in the five gather glioma cohorts.

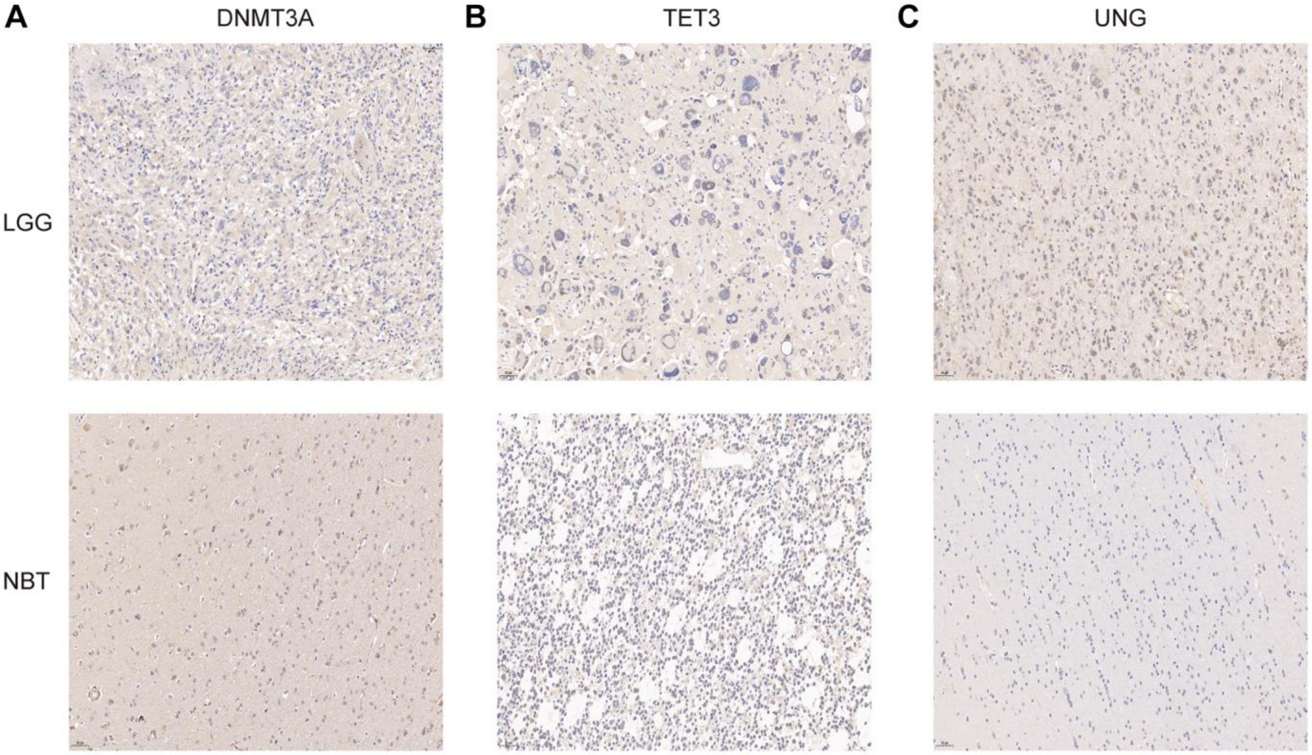

Supplementary Figure 2. Validation of the bioinformatics results using immunohistochemistry assay. Comparison of the protein expression of DNMT3A (**A**), TET3 (**B**), and UNG (**C**) in NBT and LGG by immunohistochemistry assay.

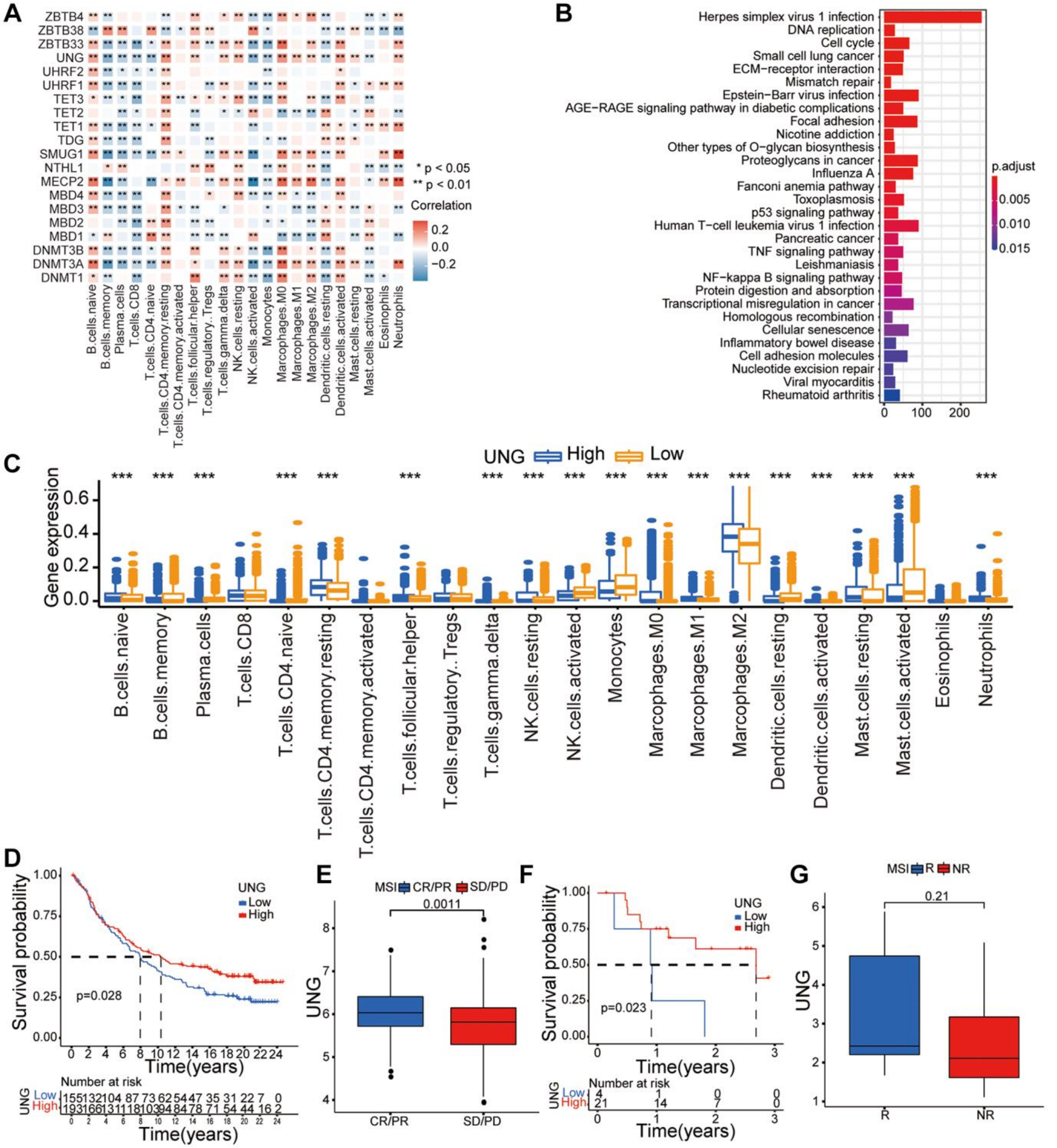

Supplementary Figure 3. Immune characteristics of 20 DNA methylation regulators and the roles of UNG in anti-PD-1/L1 immunotherapy. (**A**) The correlation between each TME infiltration cell type and each DAN methylation regulator. (**B**) KEGG analyses showed some tumor-related pathways in the UNG high-expression subgroup. (**C**) Difference in each TME infiltration cell between high and low UNG expression subgroups. (**D**, **E**) Survival analyses for patients belonging to low and high UNG expression subgroup (**D**) and differences in UNG expression among distinct anti-PD-1 clinical response groups (**E**) in the IMvigor210 cohort. (**F**, **G**) Survival analyses for patients belonging to low and high UNG expression subgroup (**F**) and differences in UNG expression among distinct anti-PD-1 clinical response groups (**G**) in the GSE78220 cohorts.

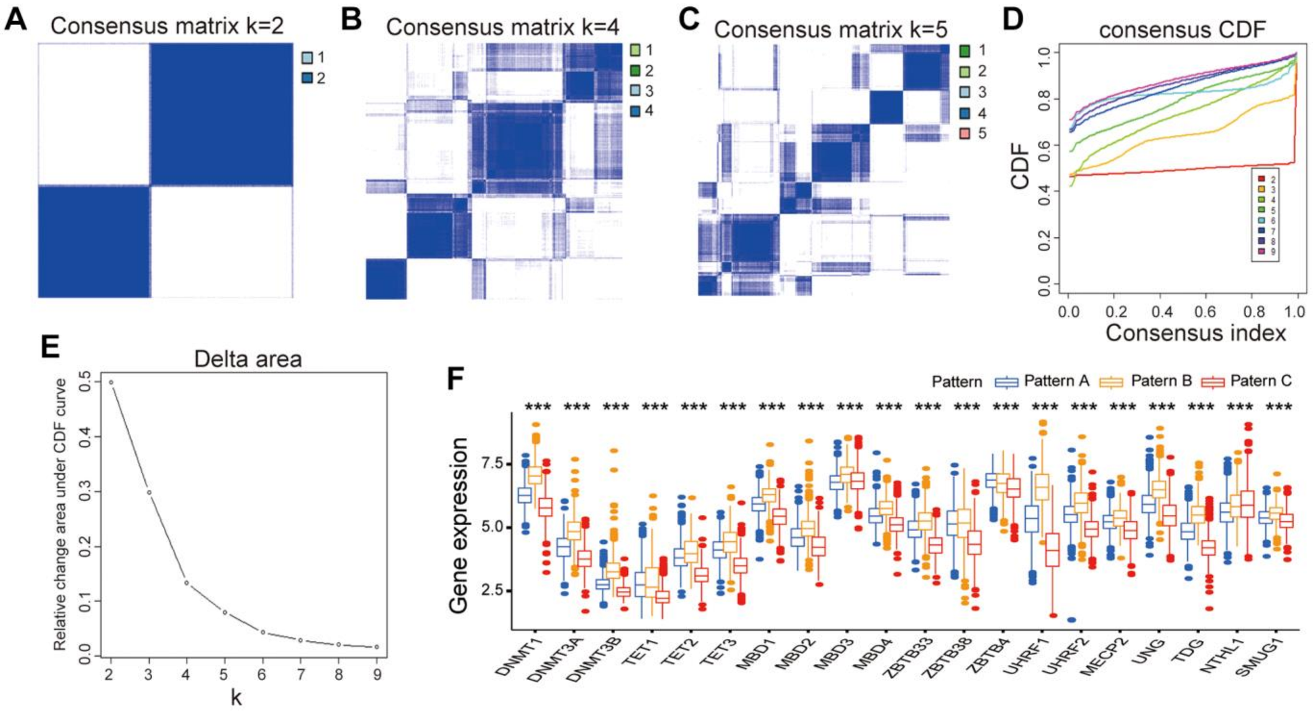

Supplementary Figure 4. Unsupervised clustering of 20 DNA methylation regulators in the gather glioma cohort. (**A**–**C**) Consensus matrices for k =2-5. (**D**) Consensus clustering CDF for k = 2–9. (**E**) Relative change in area under CDF curve for k = 2–9. (**F**) The expression of 20 regulators in the three patterns.

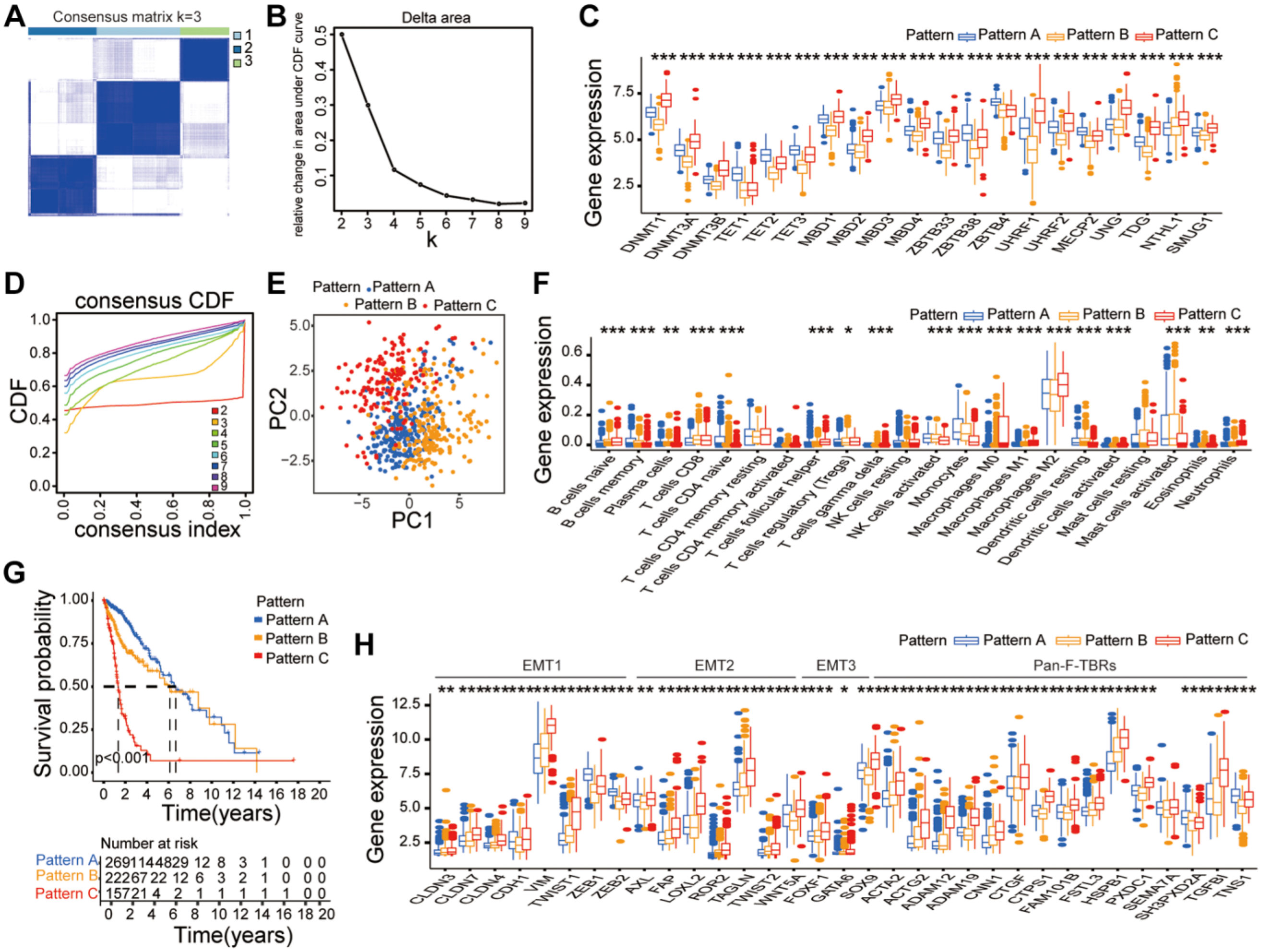

Supplementary Figure 5. Unsupervised clustering of 20 DNA methylation regulators in the TCGA datasets. (**A**) Consensus matrices of the gather glioma cohort for k =3. (**B**) Consensus clustering CDF for k = 2–9. (**C**) The expression of 20 DNA methylation regulators in the three patterns. (**D**) Relative change in area under CDF curve for k = 2-9. (**E**) PCA analysis for 20 regulators expression to the three patterns. (**F**) Difference in the expression of each TME infiltrating cell between the three patterns. (**G**) Survival analyses for patients between the three patterns. (**H**) Difference in pan-F-TBRS and the activation of EMT related genes expression between the three patterns.

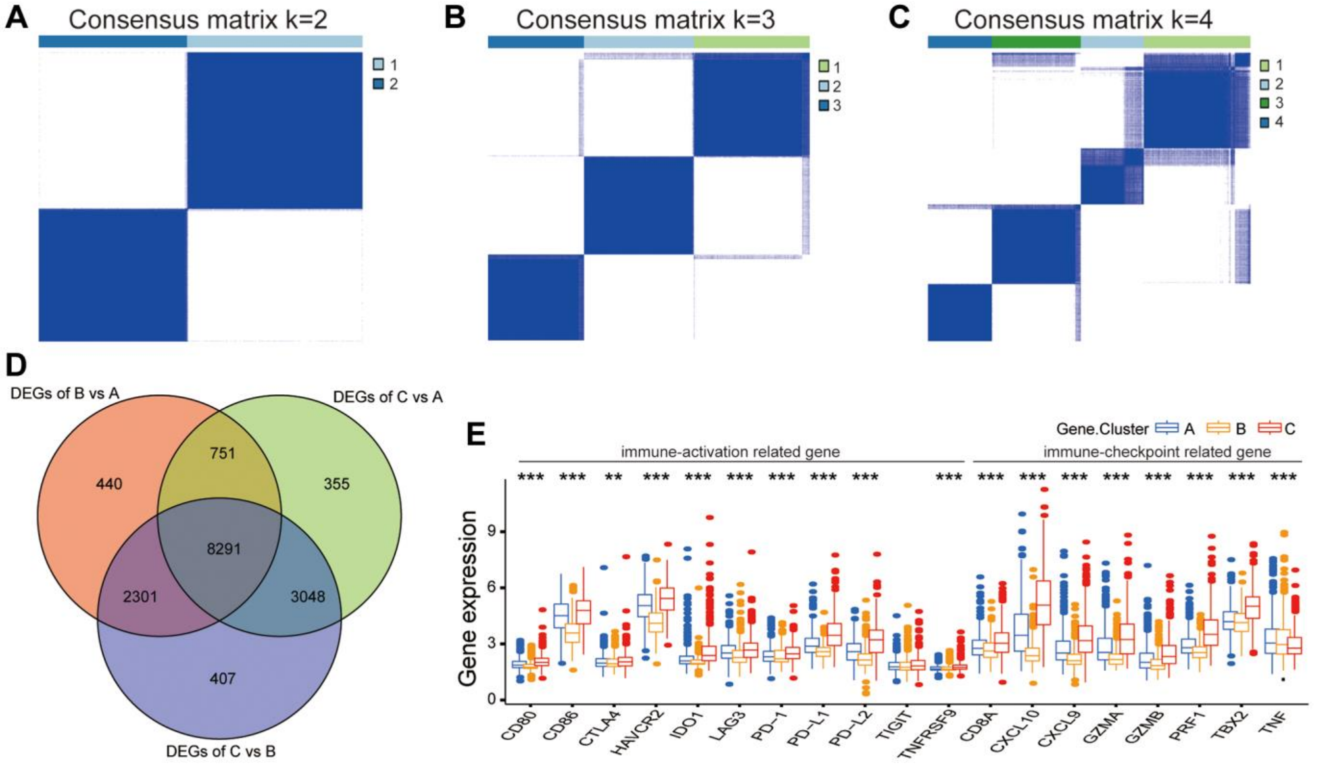

Supplementary Figure 6. Unsupervised clustering of DNA methylation related genes in the five gather glioma cohort. (**A**–**C**) Consensus matrices of the five gather glioma cohort for k =2–4. (**D**) 8291 DNA methylation related genes shown in Venn diagram. (**E**) Difference in the expression of immune activation and immune checkpoint related genes between the three gene clusters.

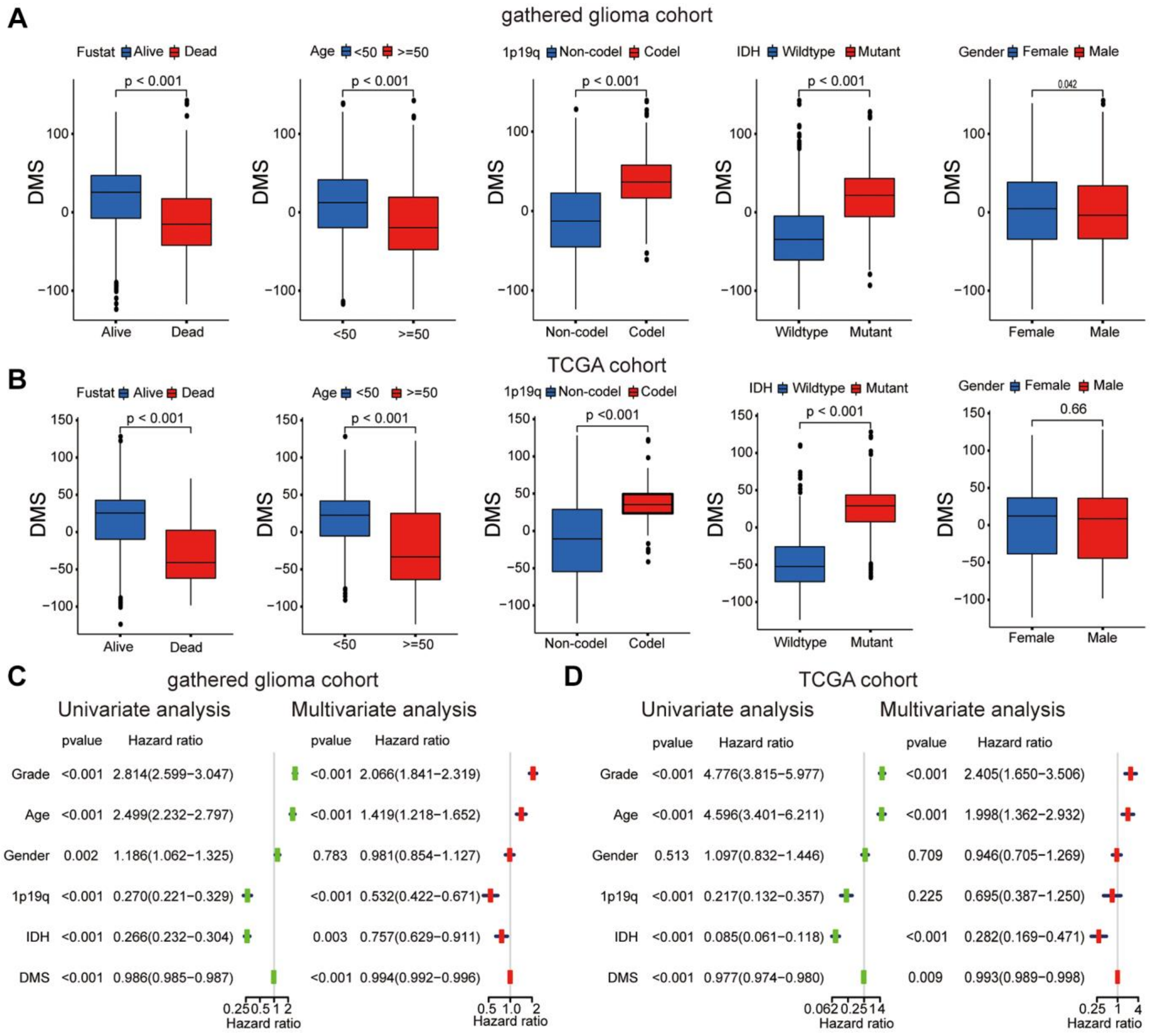

Supplementary Figure 7. Correlations between DMS and clinical characteristics in the gather glioma cohort and TCGA dataset. (A, B) Distributions of DMS stratified by fustat status, age, 1p19q status, IDH status, and gender. (**C**, **D**) Relationships between the clinical characteristics and OS of glioma patients were determined by univariate and multivariate Cox regression analyses.

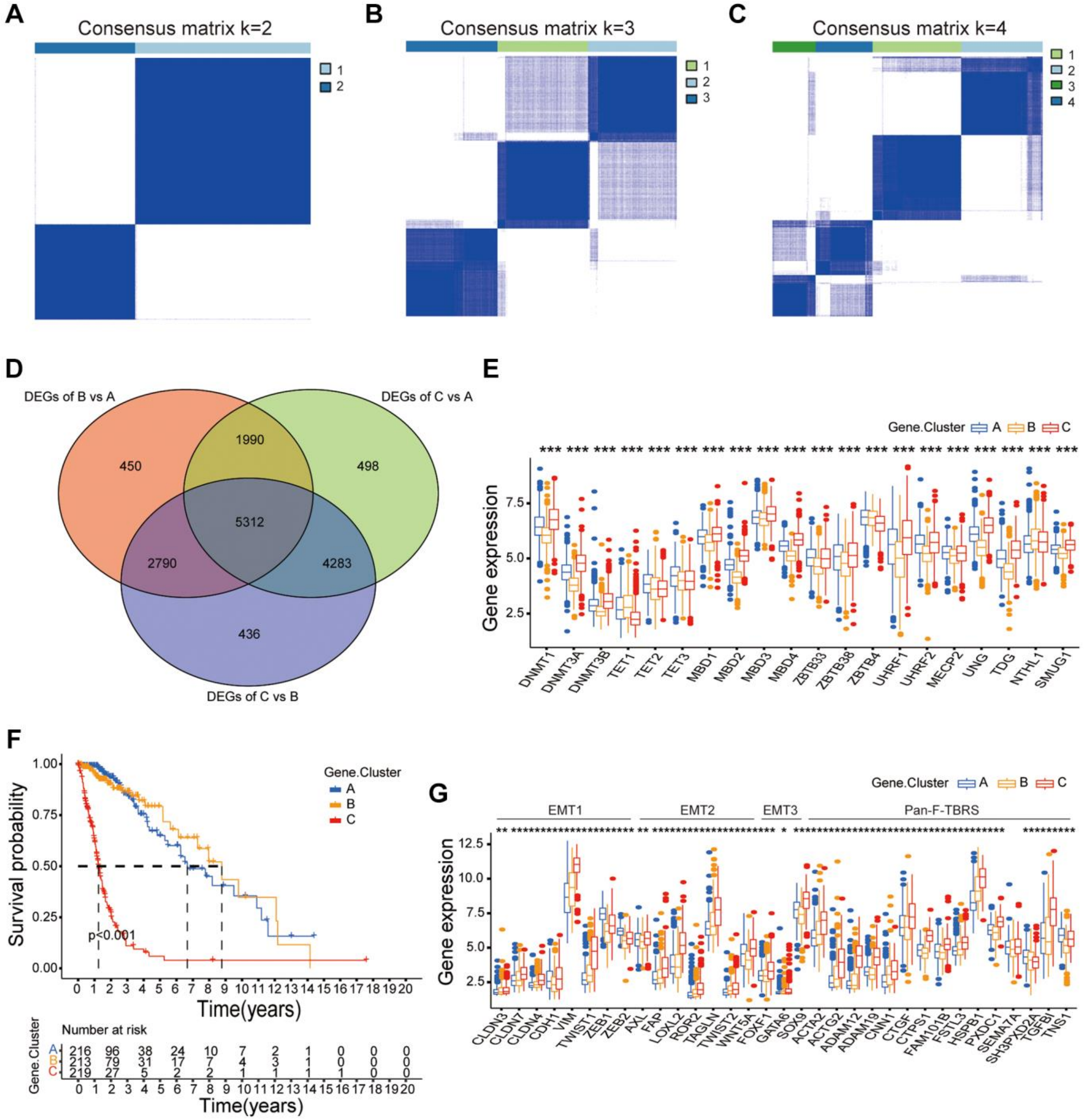

Supplementary Figure 8. Unsupervised clustering of DNA methylation related genes in the TCGA dataset. (**A**–**C**) Consensus matrices of the TCGA dataset for k =2–4. (**D**) 5312 DNA methylation related genes shown in Venn diagram. (**E**) The expression of 20 DNA methylation regulators between three gene clusters. (**F**) Survival analyses for patients between the three gene clusters. (**G**) Difference in the expression of immune activation and immune checkpoint related genes between the three gene clusters.

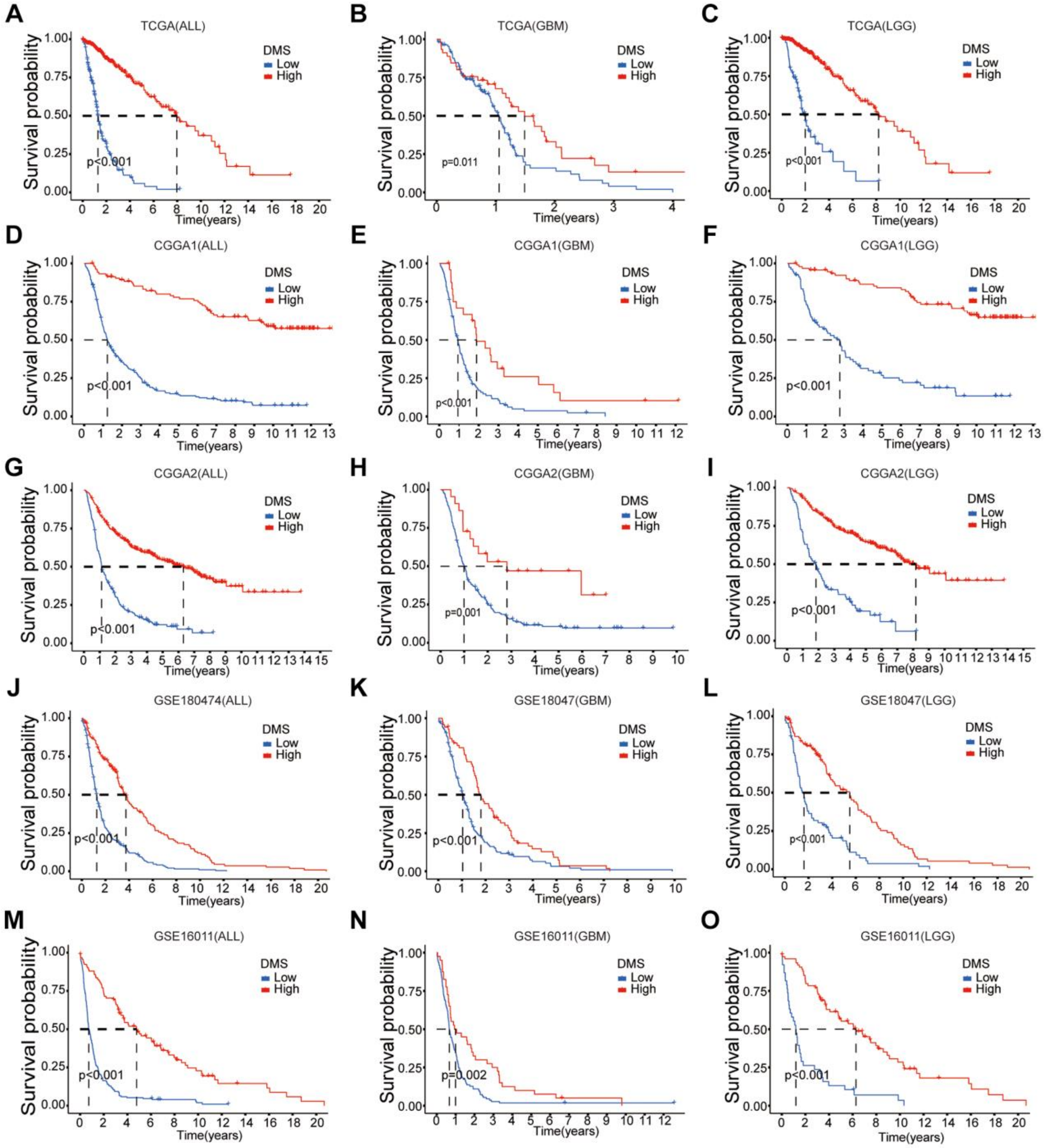

Supplementary Figure 9. Multi-cohorts survival analysis validation of DMS. Overall survival analysis of the high and low DMS groups in the CGGA1 (**A**–**C**), CGGA2 (**D**–**F**), GSE180474 (**G**–**I**), GSE16011, (**J**–**L**), and TCGA (**M**–**O**) datasets.
